# Development of a Humane Slaughter Device for Green Turtles for Use by Traditional Owners in the Torres Strait Islands, Australia

**DOI:** 10.1371/journal.pone.0167849

**Published:** 2017-01-11

**Authors:** Mark Flint, Paul C. Mills, Frank Loban, Tristan Simpson, Stan Lui, Ronald Fujii, Don Whap, Jaylene B. Flint, Helen Owen

**Affiliations:** 1 *Veterinary*-Marine Animal Research, Teaching and Investigation Unit, School of Veterinary Science, The University of Queensland, Gatton Campus, Queensland, Australia; 2 School of Forest Resources and Conservation, University of Florida, The Florida Aquarium’s Center for Conservation, Apollo Beach, Florida, United States of America; 3 Land and Sea Management Unit, Environmental Management Program, Torres Strait Regional Authority, Thursday Island, Queensland, Australia; 4 The Florida Aquarium’s Center for Conservation, Apollo Beach, Florida, United States of America; Florida State University, UNITED STATES

## Abstract

Marine turtles are caught and slaughtered for consumption as part of traditional indigenous community harvest in Australia as well as in many countries in which marine turtles can be found. However, changes to the *Animal Care and Protection Act 2001* in 2012 resulted in Australian indigenous hunters becoming potentially liable to prosecution for using traditional practices to slaughter marine turtles. To provide indigenous hunters with an alternative scientifically tested method to hunt, we developed and tested a humane method as an option to use in indigenous communities. Between 2012 and 2015, a device was developed, tested on 11 carcasses to determine effectiveness and repeatability, used on 5 anaesthetised animals independently diagnosed as candidates for euthanasia, and ultimately used on 2 healthy, conscious animals as part of normal indigenous community subsistence harvesting under observation before being left with the communities for use. Feedback was sought from the communities on the suitability and potential adoption of the device. The device effectively ablated the hind brain and severed the spinal cord when deployed in 81% (9/11) of the tested carcasses, with death in 100% (5/5) of turtles, on average, within 78 seconds of deployment on anaesthetised turtles and death in 100% (2/2) of turtles, on average, within 144 seconds when deployed on healthy turtles within community. Failure to ablate the hindbrain and sever the spinal cord in the cadaver cases was due to incorrect deployment of the device. This device showed promise as an alternative euthanasia method available to indigenous communities of the Torres Straits. Further work is required to encourage acceptance by hunters.

## Introduction

Marine turtles are caught and slaughtered for consumption as part of traditional indigenous community harvest in Australia as well as in many other countries in which marine turtles can be found.

In early March 2012, a story aired on Australian national television that questioned the traditional hunting practices and illegal trade of turtles and dugongs; two iconic species of conservation concern (http://www.abc.net.au/7.30/content/2012/s3448943.htm). The result was a public backlash causing the state of Queensland Government’s *Animal Care and Protection Act 2001* to be changed to remove the exemption from animal welfare obligations that existed for Aboriginal and Torres Strait Islander people acting according to their 4000-year old Islander custom. An offence exemption is now included to ensure that these animals when killed in the exercise of Aboriginal tradition or Island custom are killed in a way that causes *as little pain as is reasonable*. By definition, this change of wording elevated the slaughter of marine turtles to require elements of euthanasia in alignment with modern abattoir practices. Slaughter is the killing of animals for preparation of meat for human consumption, whereas euthanasia (Gk “good death”) is an easy or painless death [1].In response to this change in legislation, the Torres Strait Regional Authority (TSRA), under guidance from the Great Barrier Reef Marine Park Authority (GBRMPA) and Queensland Department of Environment and Heritage Protection (EHP), determined that a humane alternative to current traditional hunting practices to slaughter marine turtles was warranted. Such an alternative would permit hunters to continue traditional practices within the rules of the legislation.

Conventional methods for the slaughter of livestock for human consumption includes captive bolt and gunshot, electric stunning and electrocution, carbon dioxide stunning, decapitation and cervical dislocation, and ritual slaughter. Captive bolt and gunshot requires the penetration of a projectile through the skull into the brain. Non-penetrating projectiles, such as mushroom guns only deliver concussive forces and do not achieve euthanasia alone. Electric stunning and electrocution induce instantaneous unconsciousness that allow for euthanasia via exsanguination. Carbon dioxide stunning involves holding groups of animals in chambers where gas is injected to displace the oxygen resulting in anaesthetising and asphyxiation. Decapitation and cervical dislocation involve the separation of the head from the body and/or the breaking of the spinal cord to prevent sensory stimulation of the body to the brain. Ritual slaughter involves exsanguination by cutting the throat [2, 3]. In many Torres Strait Island communities, ethically-acceptable euthanasia solutions (e.g. lethal injection drugs, such as sodium pentobarbitone) and weapons, including firearms, captive bolt guns and stun guns, are restricted without special licenses and risk harm to those using them or eating meat from animals killed this way. Consequently, conventional methods are not readily available to indigenous hunters.

The right to hunt a marine turtle by Indigenous Australians is considered an earned cultural privilege within communities and only allowed when a high level of skill is achieved after years of training by elder hunters. For many of the sea faring communities, turtles and dugongs are totem animals, for which the slaughter of these animals is done with great respect. Consequently, hunters are attuned to determining a live, unconscious and dead animal as well as show a high level of empathy with their totem animal. Traditional Owner methods of slaughter vary between communities. In general, the turtle is placed on its carapace and (1) a sharp blow is delivered to the rostrum or the top of the head by a large stone when the turtle’s head is extended clear of the carapace (“stone method”); or (2) a palm frond is taken and stripped of leaves to create a sharp probe that is inserted through the nasal cavity (“broom stick method”). Both methods are simple and use freely available resources. To keep with the practicality and acceptance of these methods, a simple device with minimal moving parts was required for a viable alternative.

The proposed device would need to stabilise the skull and restrict head movement for the reliable direction of a sharp probe through the hindbrain (by ‘pithing’- the destruction of the central nervous system) and severing of the spinal cord ensuring death and ablation of any sensation or nervous communication between the body and the brain. Ablation of the hindbrain by pithing, including severing of the spine, is considered successful in achieving rapid painless death by replicating pithing and decapitation in a single function. Pithing and decapitation causing destruction of the brain and spinal cord is recommended by the American Veterinary Medical Association’s Guidelines on Euthanasia [4] as an acceptable combination method for the euthanasia of reptiles of all sizes.

Work and Balazs [5] demonstrated a sharp blow to the head with a pointed probe can effectively penetrate the skull in a small Hawaiian green turtle (*Chelonia mydas*). Considerations that need to be taken into account when using this principle to dispatch a large reptile with a solid exoskeleton are (1) the mobility of a healthy green turtle even if placed on its carapace or restrained; (2) the elasticity of the skull to percussive insults; (3) reliably being able to position the probe to ablate the hindbrain from a dorsal approach (as turtle forebrain insults are seldom fatal); (4) the need for a ‘single blow’ slaughter method to cause rapid death; (5) indigenous community restrictions; (6) indigenous and wider community perception; and (7) the need for a durable device with few moving parts that will not wear out or fail with prolonged exposure to marine elements.

The aim of this project was to establish an alternative option for the humane slaughter of marine turtles by meeting the following objectives:

1. Develop a euthanasia harness device for testing;
2. trial the harness in a step-wise manner on carcasses through to live healthy marine turtles;
3. engage the indigenous communities in trialling the device and providing feedback to its effectiveness and suitability as an alternative option to current practices.

## Materials and Methods

The University of Queensland Animal Ethics Committee Approval number: SVS/382/13/TSRA; The University of Queensland Behavioural and Social Sciences Ethical Review Project Number: 2012000895; Field Permit: Department of Environment and Heritage Protection Permit Number: WISP1199511.

### Device development

**Fig 1:**
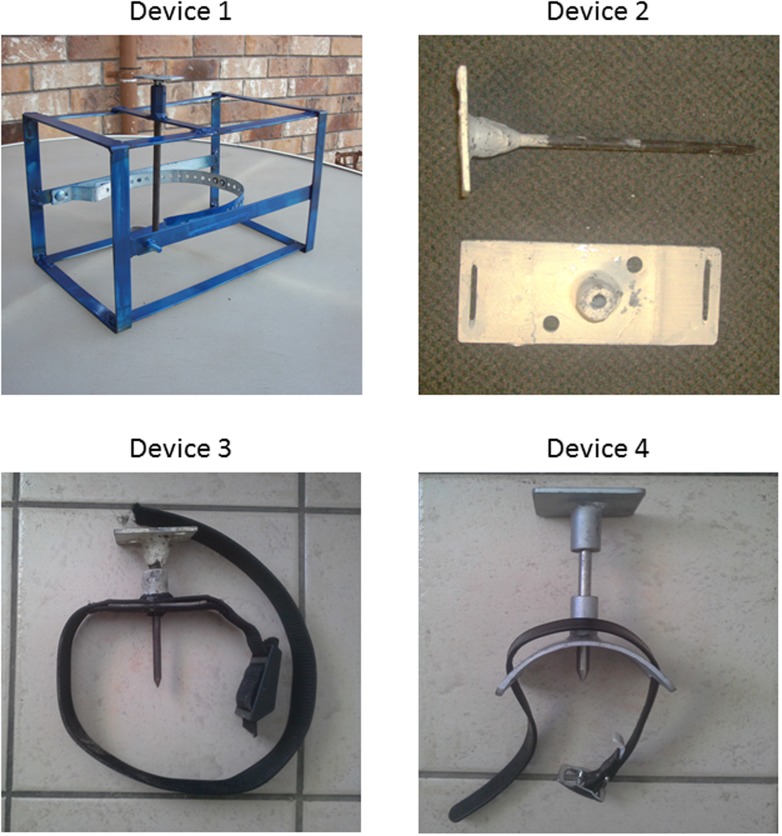
Device evolution throughout the project. All conform to the same mechanism of action.

There were four device iterations (Device 1–4) developed and tested from the first prototype in July 2012 through to the device left among the communities at the conclusion of the project between May and November 2015 (Fig 1). Each followed the same principle of penetrating the hindbrain and severing the spinal cord by introduction of a 9 mm diameter × 240 mm long probe perpendicularly through the dorsal surface of the skull at the caudal aspect of the frontoparietal scale to penetrate at least 140 mm into the adult skull to ensure severing of the spinal cord after passing through the hindbrain. Each device positioned and secured the probe with a guide placed above the frontoparietal scale, which was either a head brace, such as used in Device 1, or a head plate and strap, such as used in Device 4. Each device had an interchangeable 9 mm stainless steel probe sharpened to a rounded or chisel point at one end and a strike plate for the sledgehammer welded on the other end. As such, devices were considered the same with respect to testing the effectiveness of its use as a tool to kill marine turtles. Modifications throughout the trial were to create a more robust product and encourage community acceptance of the device. Device 4 was developed post Community feedback and was considered the most likely device to be adopted by hunters. Probe deployment was made by a single blow from a 5 kg sledgehammer “dropped” with moderate hand-operated downward force directly vertically from approximately 50 cm above the strike plate.

Device 1 was a frame prototype of a proposed enclosed box that would contain the turtle’s head and discourage movement. A head brace maintained the caudal frontoparietal scale under the probe for a range of head sizes. This maintained the strike site in the same place.

Devices 2 and 3 created a small head plate and probe guide that could be affixed to the head of the turtle immediately above the frontoparietal plate. A strap affixed the device in place.

Device 4 refined the previous devices, following trials with each device, by shortening the probe and curving the head plate to better fit the skull and stay in position.

### Carcasses

One of each iteration of the device was tested on 11 green turtle carcasses ranging in size from small juveniles through to large adults. The harness device was tested on carcasses sourced from Australian Wildlife Hospital, Queensland Parks and Wildlife Services, Underwater World (Merlin) and TSRA from the Torres Strait Island communities. All turtles were euthanased based on independent grave diagnoses or as part of a normal subsistence harvest. No capture or restraint of the carcasses were required.

Each turtle had one of the four devices described above deployed. Regardless of device, each probe deployment was identical in entry site, force and displacement. Each deployment was filmed and photos taken for assessment by the governing animal ethics committee and research analyses. The probe was carefully removed to ensure no further damage to the brain. A necropsy was performed to assess the pathway and effect of the probe entering the head. For 16 of the examined turtles, the head was opened longitudinally along the midline with the probe *in situ* to visualise the brain, spinal cord and path of the probe.

### Anaesthetised live turtles

For five turtles that were independently deemed to be candidates for euthanasia by the rehabilitation facility where they were admitted, the device and probe was deployed while under a full plane of anaesthesia. Anaesthesia was achieved by administering Alfaxalone anaesthetic solution (10 mg/mL, Jurox, Australia) at 10 mg/kg intravenously [6] via the cervical dorsal fossa. The turtle was then placed in lateral recumbency and monitored for effect of anaesthesia (loss of response to deep pain stimuli, loss of jaw tone and palpebral reflex, but maintenance of spontaneous ventilation and cardiac function). Time to reach a full plane of anaesthesia was recorded, as assessed using the above criteria.

Achieving death was determined by a veterinarian using standard assessment of loss of palpebral reflex, menace response, jaw tone, spontaneous respiration, and cardiac activity; of which the latter alone is considered non-confirmatory of death in reptiles.

For each animal, the brain was collected, preserved and assessed grossly and histologically. Success was defined as at least partially severing the spinal cord and ablating more than 50% of the hindbrain.

If the device did not achieve death with one percussive blow or the above indicators of death did not indicate death had been achieved, a euthanasia solution (sodium pentobarbitone, 325 mg/mL, Virbac, Australia at >100 mg/kg) would have been administered intravenously via the cervical dorsal fossa into the jugular vein [7]. This was not required.

### Field captured live turtles

For two live healthy turtles caught for consumption, the device was demonstrated to hunters by community members employed by TSRA while the PI was present. The harness (Device #4) was fitted and deployed as described above. The slaughter was filmed. The opportunity was given for community members to ask questions on the device and give opinions on design and the project.

Achieving of death was measured by a veterinarian using standard assessment of loss of palpebral reflex, menace response, jaw tone, spontaneous respiration, deep pain response and cardiac activity. The animals were cut up and distributed for consumption.

### Assessing success of probe deployment

A comprehensive necropsy was conducted on each turtle (head and body when available) [8]. This protocol was modified by removing the head at the C2-C3 intervertebral space, and dissecting the brain from the cranium via a midline longitudinal incision through the skull and first two cervical vertebrae, creating two hemispheres including the spinal cord through to the forebrain. The brain was carefully excised for preservation in 10% NB formalin solution for a minimum of 3 days (or until the tissue floated) to ensure complete preservation. After this time the whole brain was transversely, serially, dissected and stained with hemotoxylin and eosin. Area of remnant hindbrain (determined by anatomic location) was estimated as a proportion of hindbrain present by comparison with known hindbrain area for similar sized green marine turtles. Ablation of 50% of the hindbrain including proportion of severed spinal cord was considered successful in achieving euthanasia by replicating pithing and decapitation in a single function. Pithing and decapitation causing destruction of the brain and spinal cord is recommended by the American Veterinary Medical Association (AVMA) Guidelines on Euthanasia [4] as an acceptable combination method for the euthanasia of reptiles of all sizes.

### Indigenous community engagement and feedback

Prior to live animal demonstrations, each participating community was visited to discuss the project in an open forum and gain feedback. This was reinforced with local ranger discussions prior to another visit to demonstrate the device that had been created based on community feedback, and then by demonstrations on carcases and live turtles. Each visit was supported by community announcements in the weeks leading up to the visit and by information sheets left at the meeting. After demonstrations on the live turtles to each community, at least one device was left with a community official to be made available to hunters to trial for approximately 6 months. After this time, TSRA officers contacted each device controller and asked for the feedback of the community. Summaries were dictated to the TSRA who provided these texts for assessment. Findings are presented as paraphrasing. Conclusions based on these opinions were weighted and drawn.

## Results

### Carcasses

**Fig 2:**
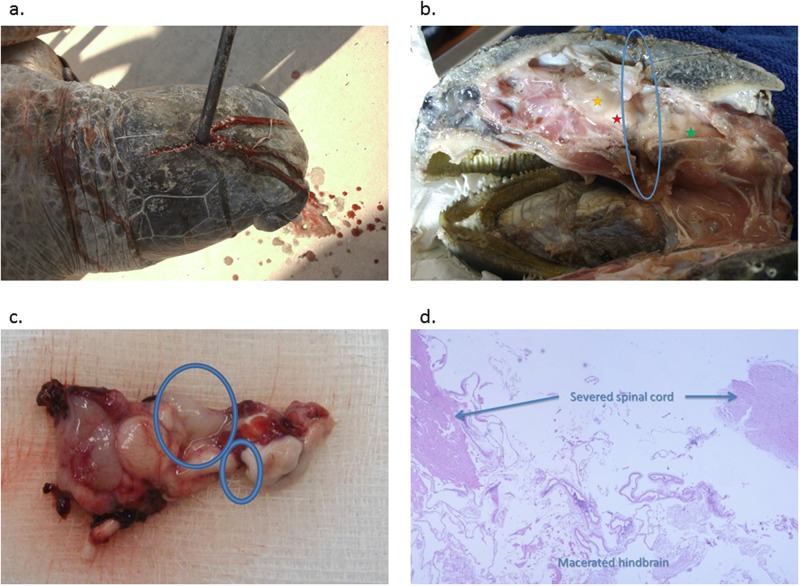
Device deployment, path of insertion and resultant destruction of the brain and spinal cord. Typical (A) presentation for correct penetration of the device through the skull at the caudal aspect of the frontoparietal scale; (B) longitudinal section showing passage of probe (encircled) with severed spinal cord (green) and ablated hindbrain with fore (yellow) and mid (red) brain intact; (C) gross appearance of a brain that has successfully had the device deployed where the hindbrain is ablated (large circle) and spinal cord (small circle) is severed; and (D) the resultant histology showing separation of the spinal cord (arrows) and the disrupted architecture of the hindbrain.

For the 11 carcasses on which the devices were deployed, this method was considered successful for nine carcasses (81%, 9/11; 95% CI 48.2–97.7; Fig 2). For the two failures, in both cases the probe did not penetrate the cranial case, rather deflected off to the side within the skull.

### Live turtles

Five turtles were anaesthetised prior to device deployment and two were slaughtered by local indigenous hunters in country while the researcher was present as part of the device trails. In all cases, deployment was measured as successful with an average of 96.7 seconds (10 seconds to 180 seconds) from deployment to confirmed death (Table 1). Where gross examination and histology were performed on the brain, findings were consistent with those reported for probe deployment on the carcasses.

**Table 1:** Duration from deployment of device to confirmed death of the turtle in live turtles.

Turtle	Death Time (s)	Anaesthetised
1	180	Y
2	10	Y
3	180	Y
4	10	Y
5	10	Y
6	107	N
7	180	N

Death was confirmed by absence of respiration, deep pain reflex and palpebral reflex. Heart beat was considered a secondary assessment tool.

### Gross examination

A successful deployment penetrated the suture of the parietal plates (Fig 2a) passing through the thickened cranial projection of these plates into the caudal braincase and rostral portion of the spinal canal and down into the pterygoid. The path can be followed as in Fig 2b resulting in gross disruption of the central nervous tissue as seen in Fig 2c. Misfiring of the probe resulted in penetration of the suture of the parietal plates but deflection laterally into the intracranial space on either side of the braincase.

### Microscopic examination

Histology was performed on 11 of the 18 turtles examined (all except seven live turtles- five euthanased through a rehabilitation centre and two from the participating indigenous communities). In all cases, histology confirmed whether the probe had successfully hit the target tissues and the degree of tissue damage (S1–S16 Figs). Tissue damage was denoted by the removal of tissues, remnant tissues showing evidence of tearing, with possible presence of bleeding and other signs of trauma (Fig 2d). All hindbrains and spinal cords were at least 50% destroyed in the slaughtered turtles.

### Community feedback

After the community demonstrations on the live turtles, the device was left with the three islands of community hunters and/or rangers for use within the community over the next 6 months.

#### Mabuiag

hunters used the device twice. They felt it was not as effective as traditional methods with respect to instant death, the device was hard to fit to the turtle head and it did not accommodate the range of turtle sizes they harvested. More education and technical support on the use of the device was requested.

#### Masig

hunters used the device. They understood the issues that surround the way marine turtles are being traditionally killed and were supportive of the harness project trials. They expressed design concerns including the number of people required to deploy the device (three people vs. usual single hunter), probe thickness and sharpness, securing the device to the head, and need for adjustment of the plate moulding.

#### Badu

hunters used the device at least twice. Like the Masig hunters, they were aware of the negative publicity that surrounded marine turtle hunting and therefore Traditional Owners/ hunters were willing to support the trials of the harness project and work towards improving the situation for communities. They expressed similar design concerns as the Masig hunters regarding probe thickness, securing the device to the head, the fact that it did not fit all turtle sizes, and the need for 2–3 people to deploy the device.

## Discussion

This research met the aim to establish an alternative option for the humane slaughter of marine turtles for the participating Torres Strait Island Communities. It also met each of its three objectives: (1) We developed an effective euthanasia harness device; (2) We trialled the harness in a step-wise manner on carcasses through to live healthy marine turtles to ensure a robust effective design that repeatedly ablated the hindbrain and severed the spinal cord when deployed correctly; (3) We engaged the indigenous communities in developing and trialling the device through visits and feedback in an attempt to develop a device with indigenous input, ownership and thus optimise the likelihood of community acceptance.

Over 3.5 years of testing, we built on the preliminary study conducted by our colleagues in Hawaii [5] and developed a device capable of penetrating the skull using a 9 mm stainless steel probe over the caudal aspect of the frontoparietal scale. When correctly positioned and deployed with sufficient force, the probe was able to pass through the caudal aspect of the brain and effectively ablate the hindbrain and sever the spinal cord (Fig 1), rendering death. In reptiles, it is inherently difficult to confirm death. We employed the loss of palpebral reflex, menace response, jaw tone, spontaneous respiration, deep pain and cardiac activity as indicators of death. It is common for cardiac function to cease and spontaneously regenerate several minutes later in many reptile species under duress or persist in reptiles even after removal from the body. As such, presence or absence of a heart beat was not considered conclusive in death. Cellular death (vs prolonged loss of consciousness) is facilitated by pithing [4], which the device tested in these trials achieves by the probe ablating the hindbrain. Traditional Owner slaughter practice also facilitates this process of death as turtles are immediately fully bled (achieving exsanguination) so the blood can be collected before it congeals, and the carcass butchered.

In this study, histology was limited to confirming that target structures had been destroyed and the extent of destruction (Fig 2d). From the live animals that were successfully euthanized while under anaesthesia, it was shown that death occurred even if only 50% of the hindbrain and spinal cord was disrupted. Minimal tissue destruction requirements were not determined, but at least 50% disruption is fatal.

The device successfully achieved euthanasia via two mechanisms. (1) Ablating the hindbrain and (2) severing the spinal cord. Ablation is the equivalent of pithing which is the destruction of neural tissue rendering the recipient to have no cognitive function or feel pain [1]. Severing the spinal cord is the nervous equivalent to decapitation (removal of the head) [1], whereby the organs are deprived of all involuntary functions. Ablation (pithing) and decapitation comply with international standards for humane euthanasia in a large reptile [4].

These methods should be suitable for use in food animals, with minimal production and systemic dissemination of brain emboli, and rapid achieving of death. Although pithing is not recommended in animals intended for human consumption due to risk of embolism of brain tissue containing specified risk material into the lungs or muscles that are eaten [9], there is no known specified risk material (e.g. bovine spongiform encephalitis prions in cattle) in a wild caught healthy marine turtles of the Torres Strait. When deployed on live animals, the device was effective in causing death between 10 seconds and 3 minutes (Table 1). This is within acceptable timeframes identified in slaughter of livestock [2].

Although capture, restraint and traditional slaughter were untested in this research, traditional slaughter methods may have merit as a humane hunting practice. The alternatives to this proposed device are the stone and broomstick methods. These methods have been long used by hunters within the Torres Strait communities and are reported to be effective methods of slaughter. They may cause shock to the cranial and peripheral nerves associated with the head causing a sensory overload to the brain and result in loss of consciousness allowing for slaughter to be humanely performed by exsanguination. In this respect, these methods may be a variance of the standard electrical stunning that results in instantaneous loss of consciousness and tonic-clonic muscular movement. These practices are used in a range of species of different sizes in abattoirs throughout the world [2].

Limitations to this device were noted during the carcass and live turtle phases. For successful deployment of the device, it needed to be positioned correctly, both over the right place on the skull but also deployed at the right angle (perpendicular to the top of the skull in both planes). To maintain this position, time was required to fit the device correctly. When this was not achieved, the probe missed the hindbrain and spinal cord. While device fitting was easily achieved under laboratory conditions, it was more of a challenge in healthy, active animals and a negative experience for indigenous hunters. Better design to secure the device such as a head-clamp is required. If the device misfires and does not achieve hindbrain ablation and spinal cord severing in one percussive blow, it is recommended the device be immediately redeployed. Alternative traditional methods may be of use under these circumstances, but cannot be recommended until they are tested. Improvements to the device design could include a single fitting piece that secures the head plate to the skull such as a flexible clamp and a handle to allow control of the head once the device is secured. Any modifications should be done in consultation with communities and consider the harshness of the marine environment to moving parts and durability.

Other negative feedback from the communities were concerns about the need and design of the device including usability in country and concerns over loss of traditional practice, repercussions of project outcomes and preference to use their traditional methods over the device. Hunters felt traditional methods were equally humane and more than one person was required to use the device. However, they expressed a willingness to continue to trial and refine the device to create an agreeable design that can be used by a single hunter to humanely slaughter a marine turtle in a quick and effective manner and comply with changing legislation.

An objective of this project was not to develop a device to replace existing traditional methods, but to provide an alternative method for optional adoption. The next step in this investigation should include ongoing work with the communities to produce a device that is acceptable to the hunters and encourage adoption. Once this has been achieved, support for use by managers and use by government staff as an ancillary tool for marine turtle euthanasia may benefit the wider community. Consultation is needed to define the requirements for endorsement by government and veterinary officials, including acceptance as part of standard reptile euthanasia guidelines and slaughter guides [3, 4]; although this device has so far met the standards outlined by welfare guides.

## Conclusion

The device developed severs the spinal cord and ablates the hindbrain in a manner that is in accordance with recommendations by the AVMA Guidelines on euthanasia for reptiles and would achieve the requirements of the *Animal Care and Protection Act 2001* to cause as little pain as is reasonable. Indigenous communities were supportive of the device although there will be much needed roll-out, including modification, further trialling and extensive communication, before the alternative device is preferred to traditional hunting practices. There is no governmental requirement to investigate currently-used traditional hunting methods or for hunters to use the device we present here.

## Supporting Information

S1 Fig Histological cross section of hind brain and spinal cord in Turtle 1A. H&E 10x.
(PDF)

S2 Fig Histological cross section of hind brain and spinal cord in Turtle 1B. H&E 10x.
(PDF)

S3 Fig Histological cross section of hind brain and spinal cord in Turtle 2A. H&E 10x.
(PDF)

S4 Fig Histological cross section of hind brain and spinal cord in Turtle 3A. H&E 10x.
(PDF)

S5 Fig Histological cross section of hind brain and spinal cord in Turtle 3B. H&E 10x.
(PDF)

S6 Fig Histological cross section of hind brain and spinal cord in Turtle 4A. H&E 10x.
(PDF)

S7 Fig Histological cross section of hind brain and spinal cord in Turtle 4B. H&E 10x.
(PDF)

S8 Fig Histological cross section of hind brain and spinal cord in Turtle 5A. H&E 10x.
(PDF)

S9 Fig Histological cross section of hind brain and spinal cord in Turtle 5B. H&E 10x.
(PDF)

S10 Fig Histological cross section of hind brain and spinal cord in Turtle 6A. H&E 10x.
(PDF)

S11 Fig Histological cross section of hind brain and spinal cord in Turtle 7A. H&E 10x.
(PDF)

S12 Fig Histological cross section of hind brain and spinal cord in Turtle 8A. H&E 10x.
(PDF)

S13 Fig Histological cross section of hind brain and spinal cord in Turtle 9A. H&E 10x.
(PDF)

S14 Fig Histological cross section of hind brain and spinal cord in Turtle 10A. H&E 10x.
(PDF)

S15 Fig Histological cross section of hind brain and spinal cord in Turtle 10B. H&E 10x.
(PDF)

S16 Fig Summarized histological cross section of hind brains and spinal cords in Turtles 1 to 11. H&E 10x.
(PDF)
