# Supplementary figures and images for: Development of a Humane Slaughter Device for Green Turtles for Use by Traditional Owners in the Torres Strait Islands, Australia
Source: PLoS One. 2017 Jan 11;12(1):e0167849. doi: 10.1371/journal.pone.0167849 (PMC5226787; doi:10.1371/journal.pone.0167849)

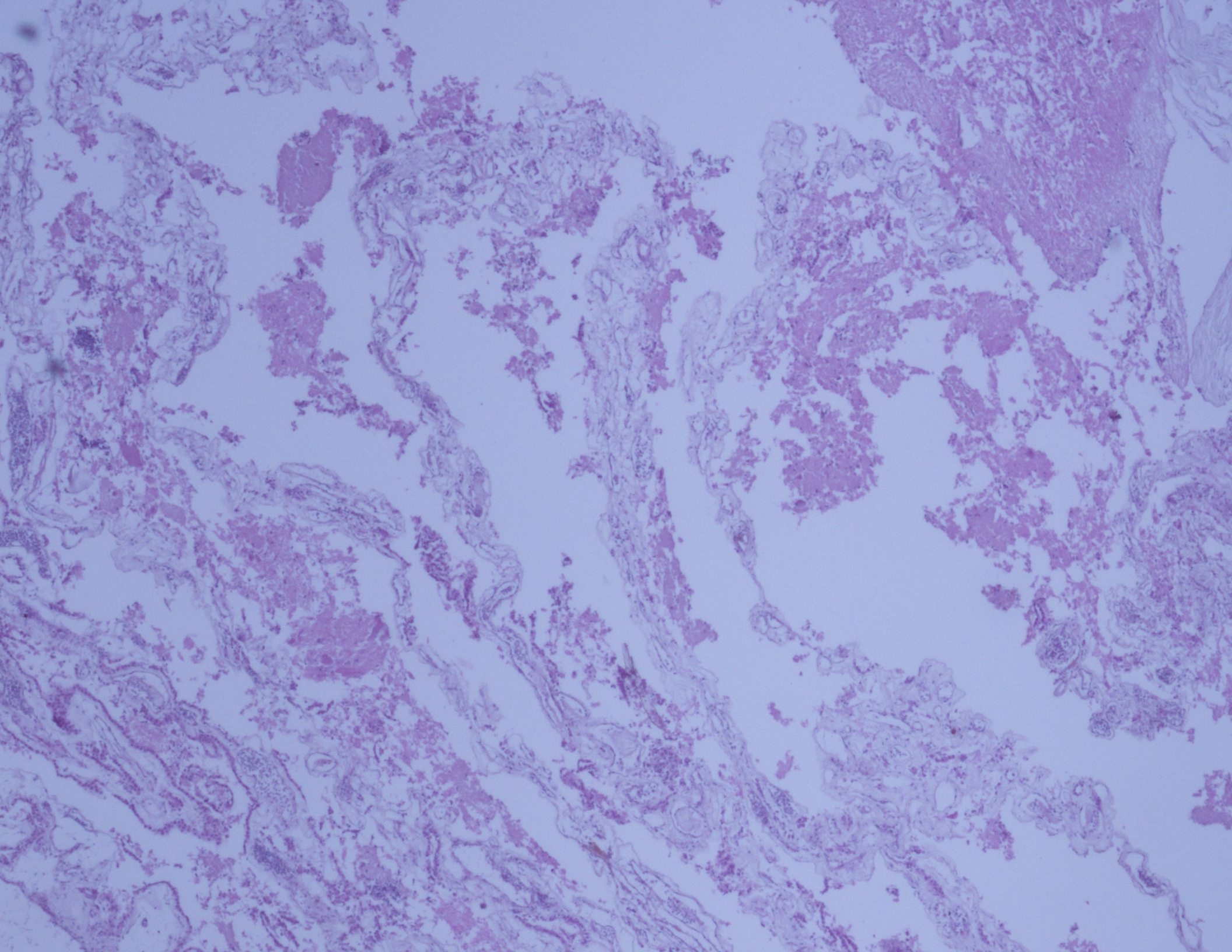

Supplement: S1 Fig — H&E 10x. (PDF) [file pone.0167849.s001.pdf]

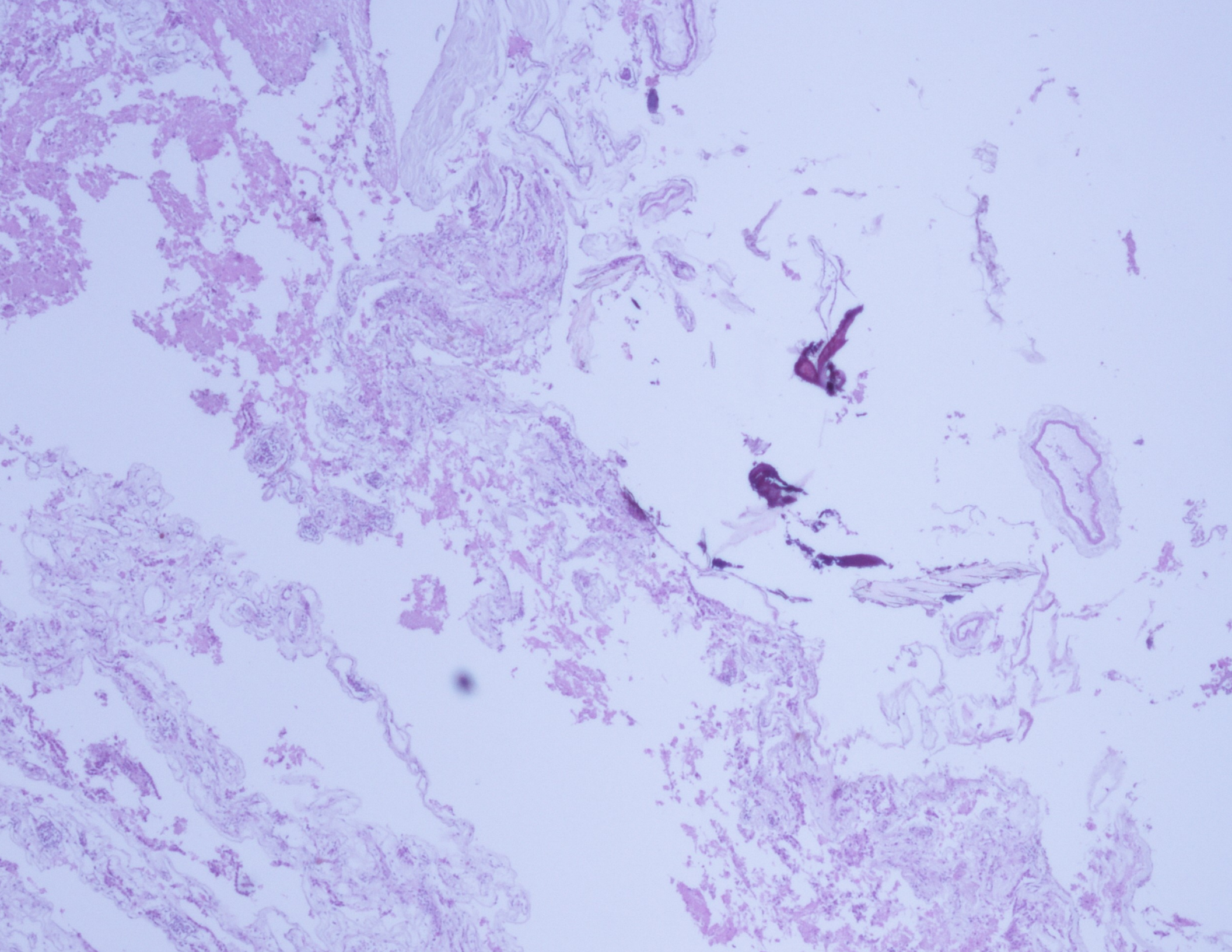

Supplement: S2 Fig — H&E 10x. (PDF) [file pone.0167849.s002.pdf]

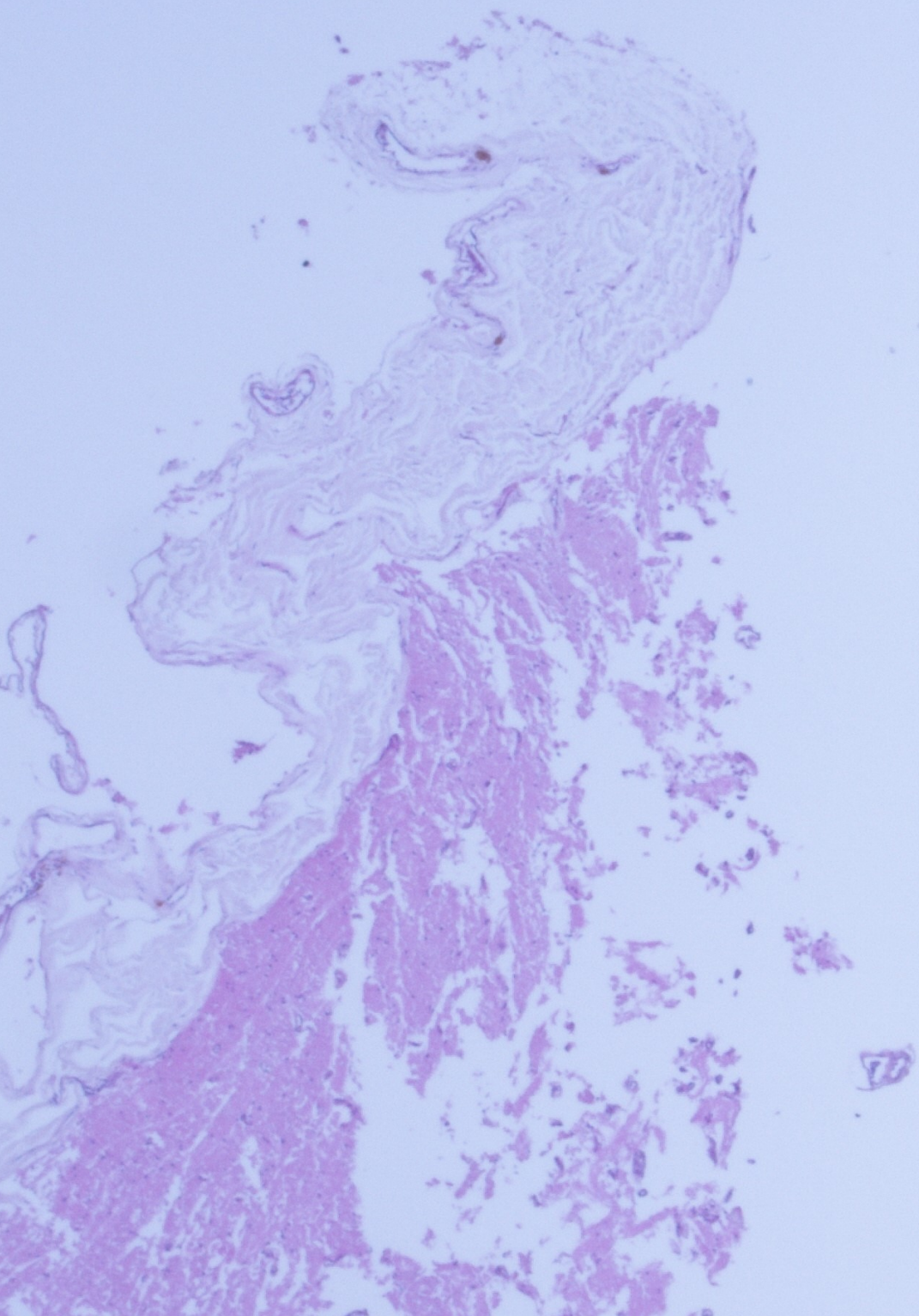

Supplement: S3 Fig — H&E 10x. (PDF) [file pone.0167849.s003.pdf]

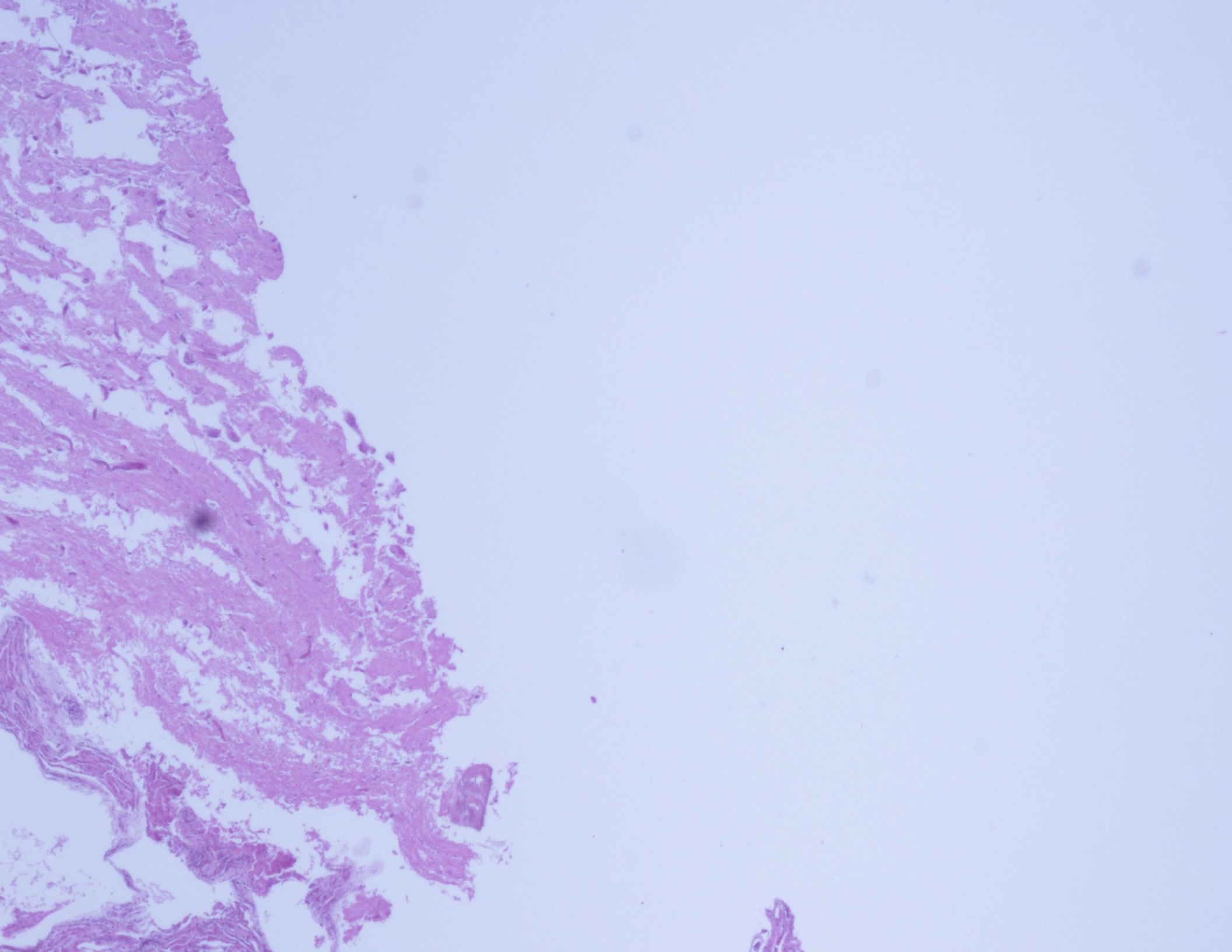

Supplement: S4 Fig — H&E 10x. (PDF) [file pone.0167849.s004.pdf]

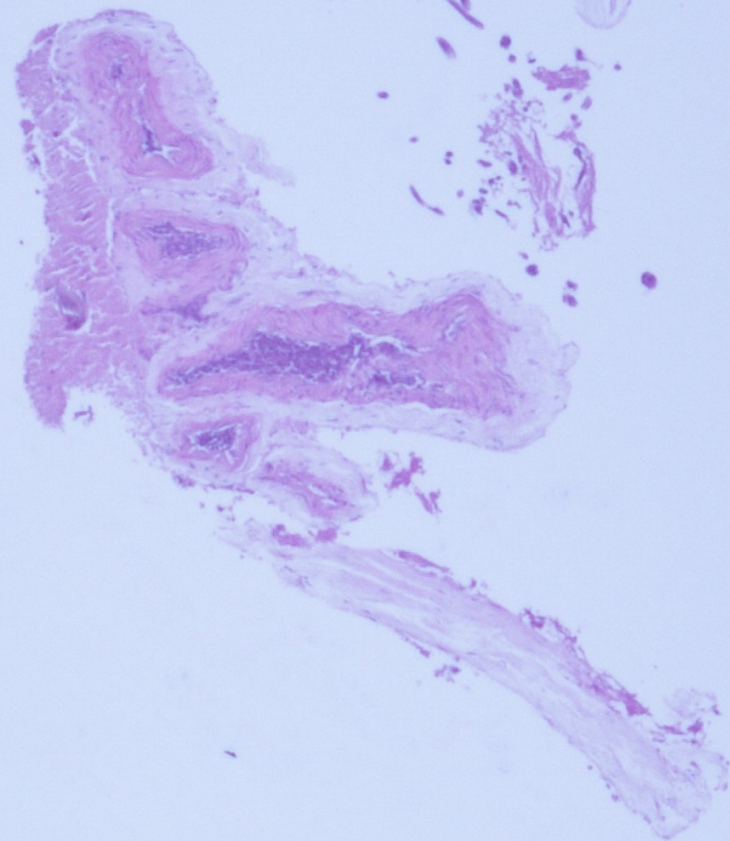

Supplement: S5 Fig — H&E 10x. (PDF) [file pone.0167849.s005.pdf]

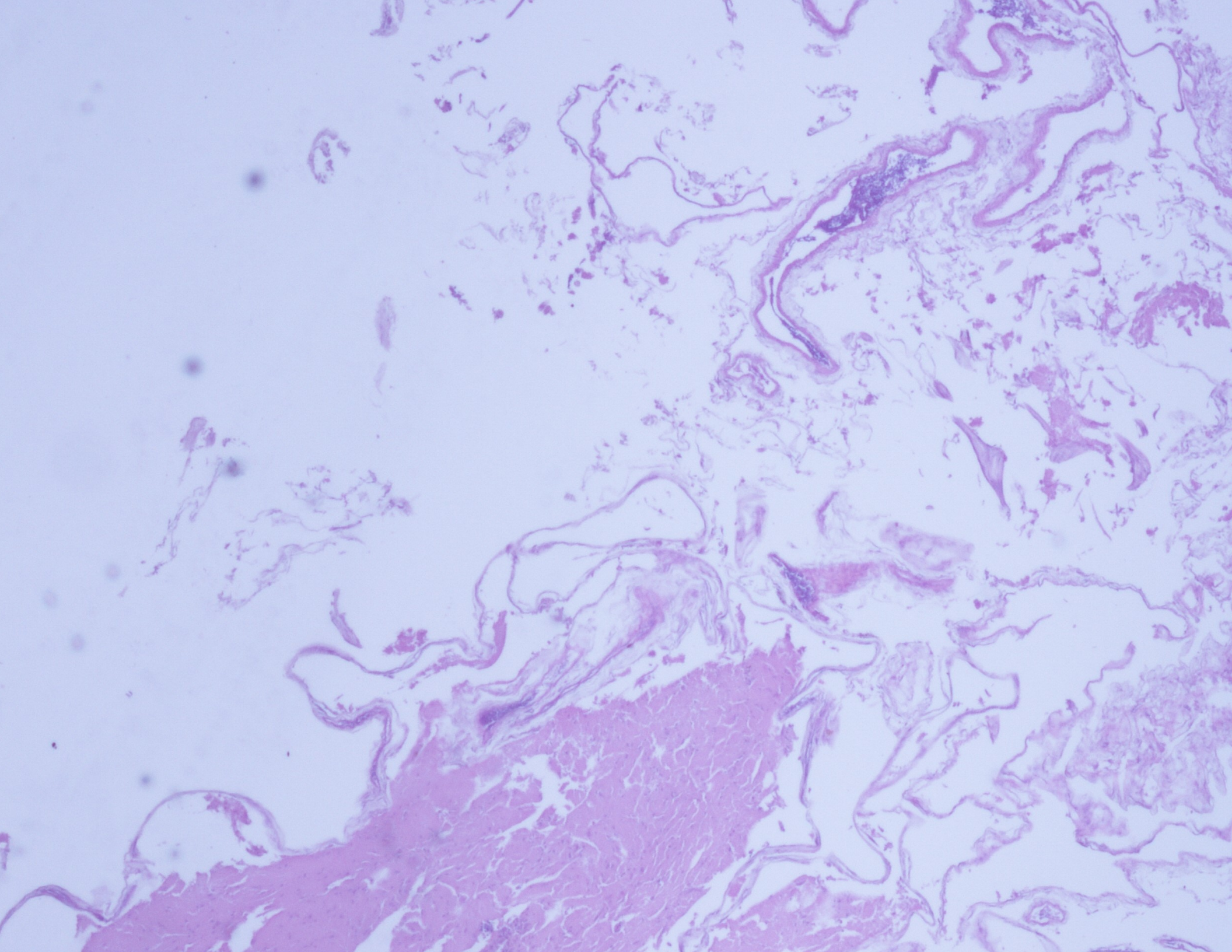

Supplement: S6 Fig — H&E 10x. (PDF) [file pone.0167849.s006.pdf]

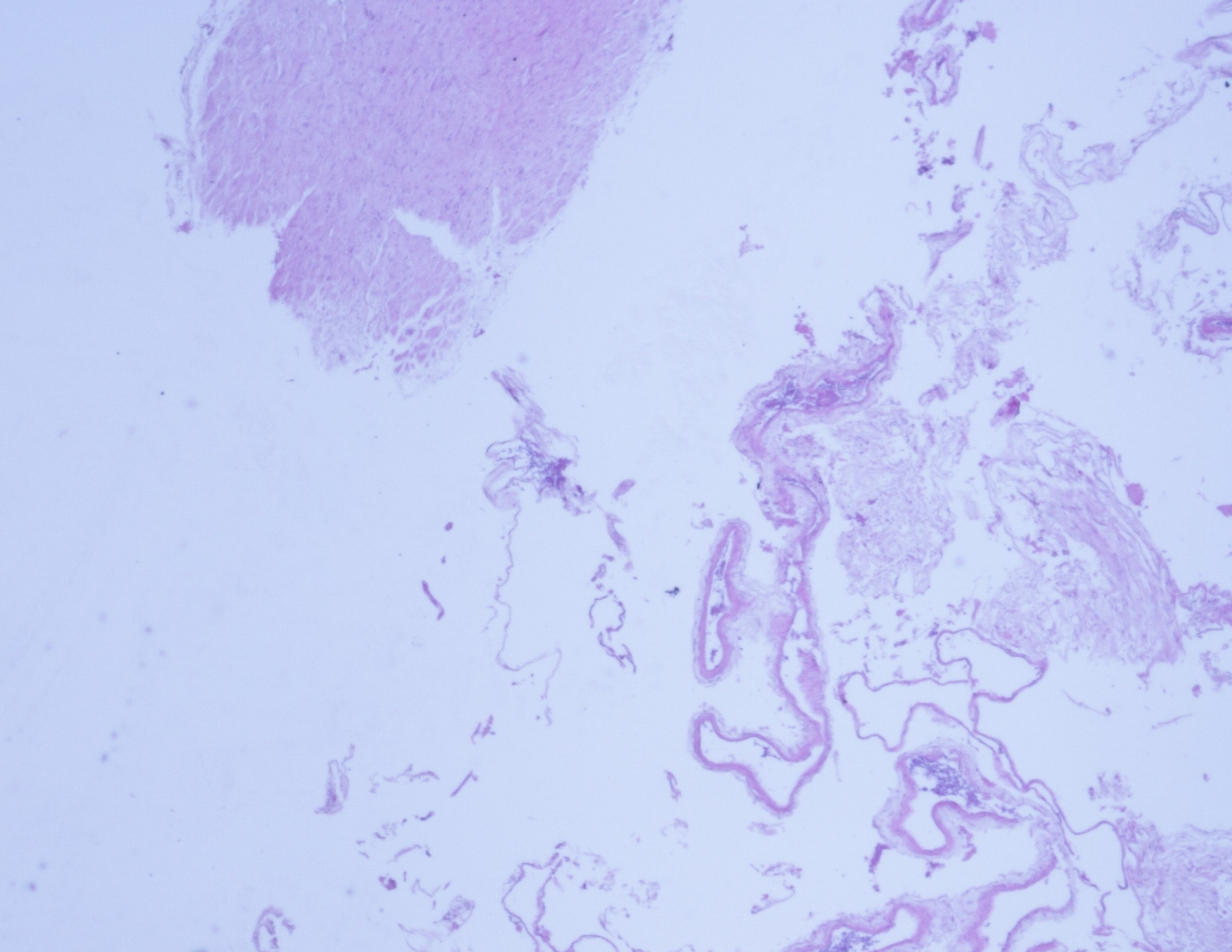

Supplement: S7 Fig — H&E 10x. (PDF) [file pone.0167849.s007.pdf]

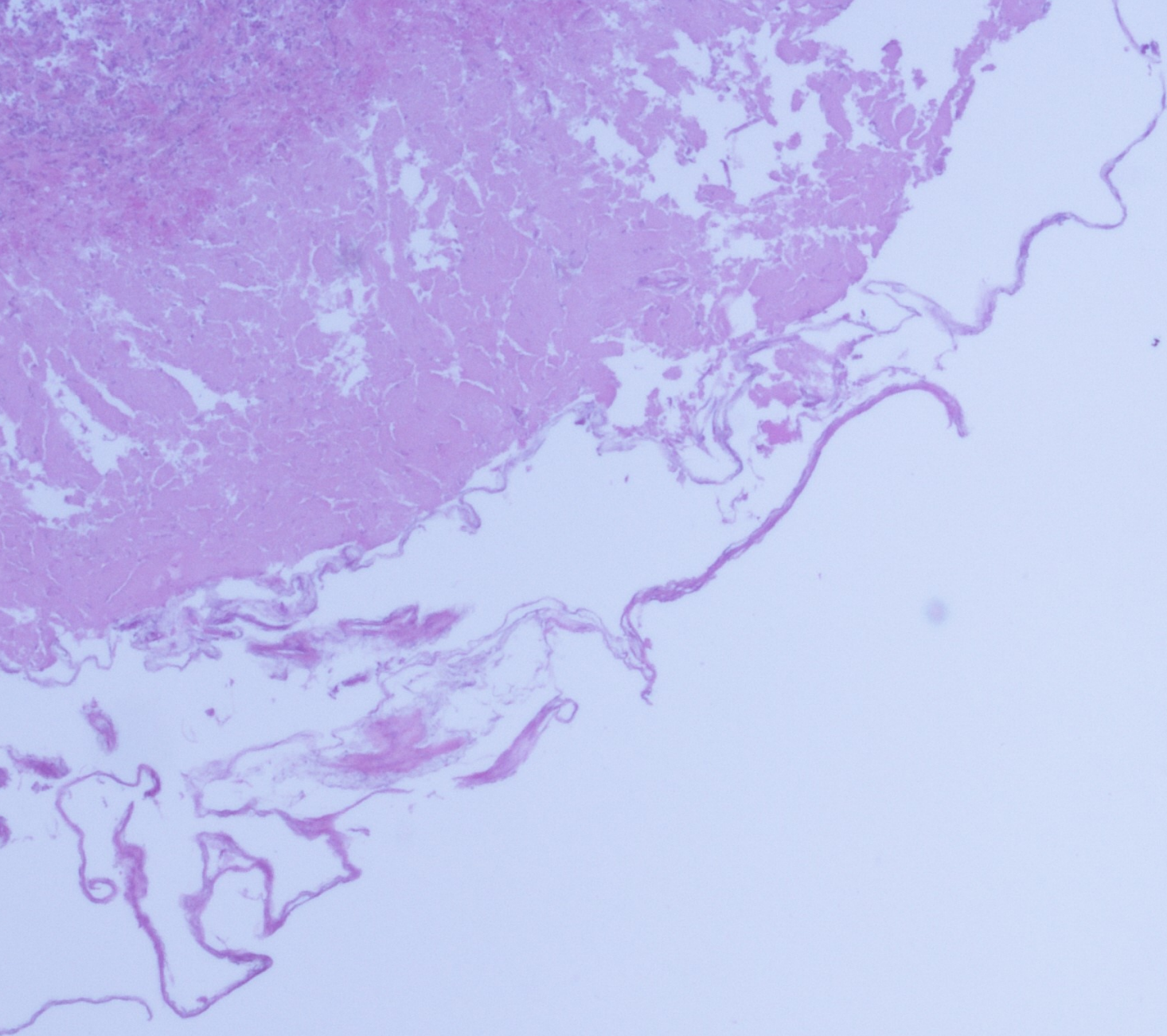

Supplement: S8 Fig — H&E 10x. (PDF) [file pone.0167849.s008.pdf]

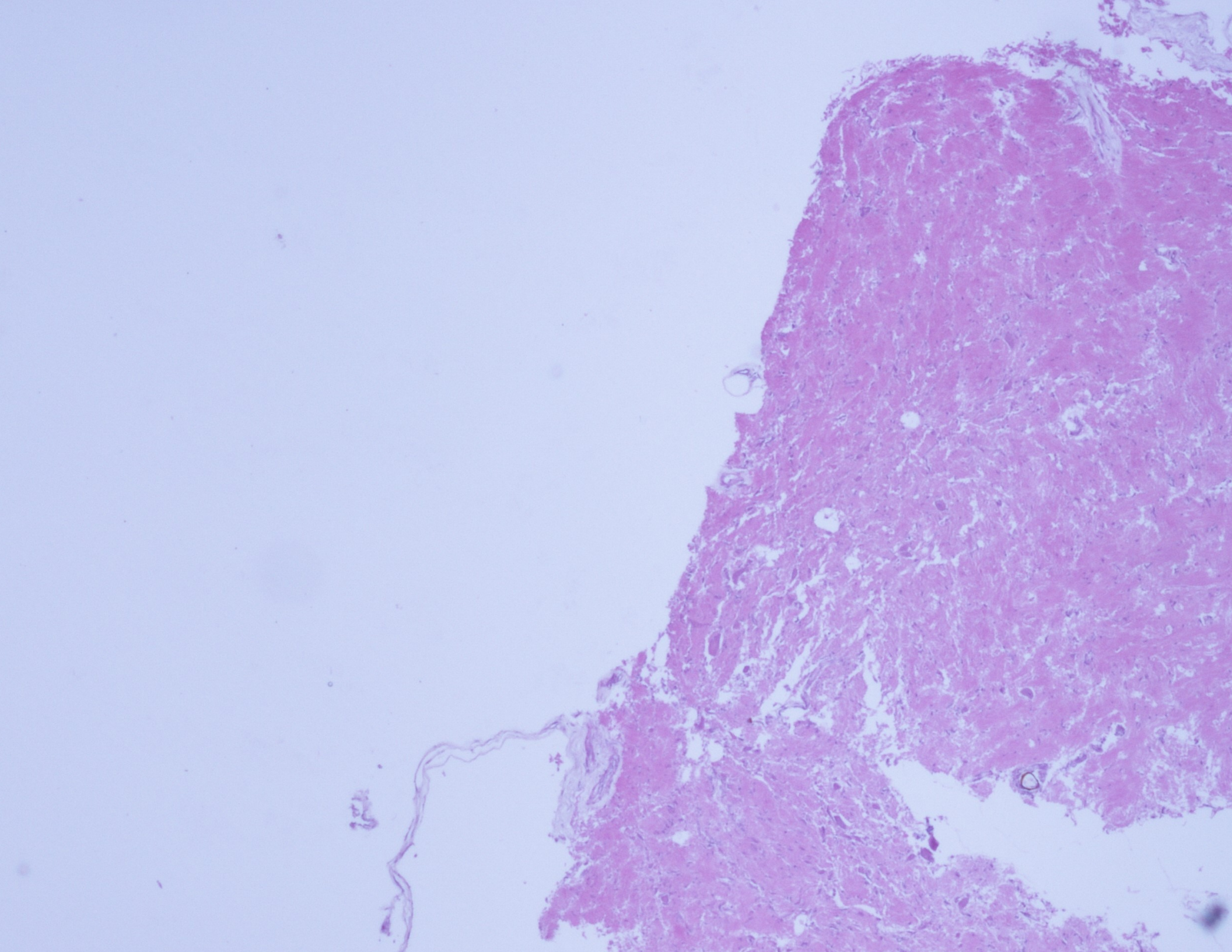

Supplement: S9 Fig — H&E 10x. (PDF) [file pone.0167849.s009.pdf]

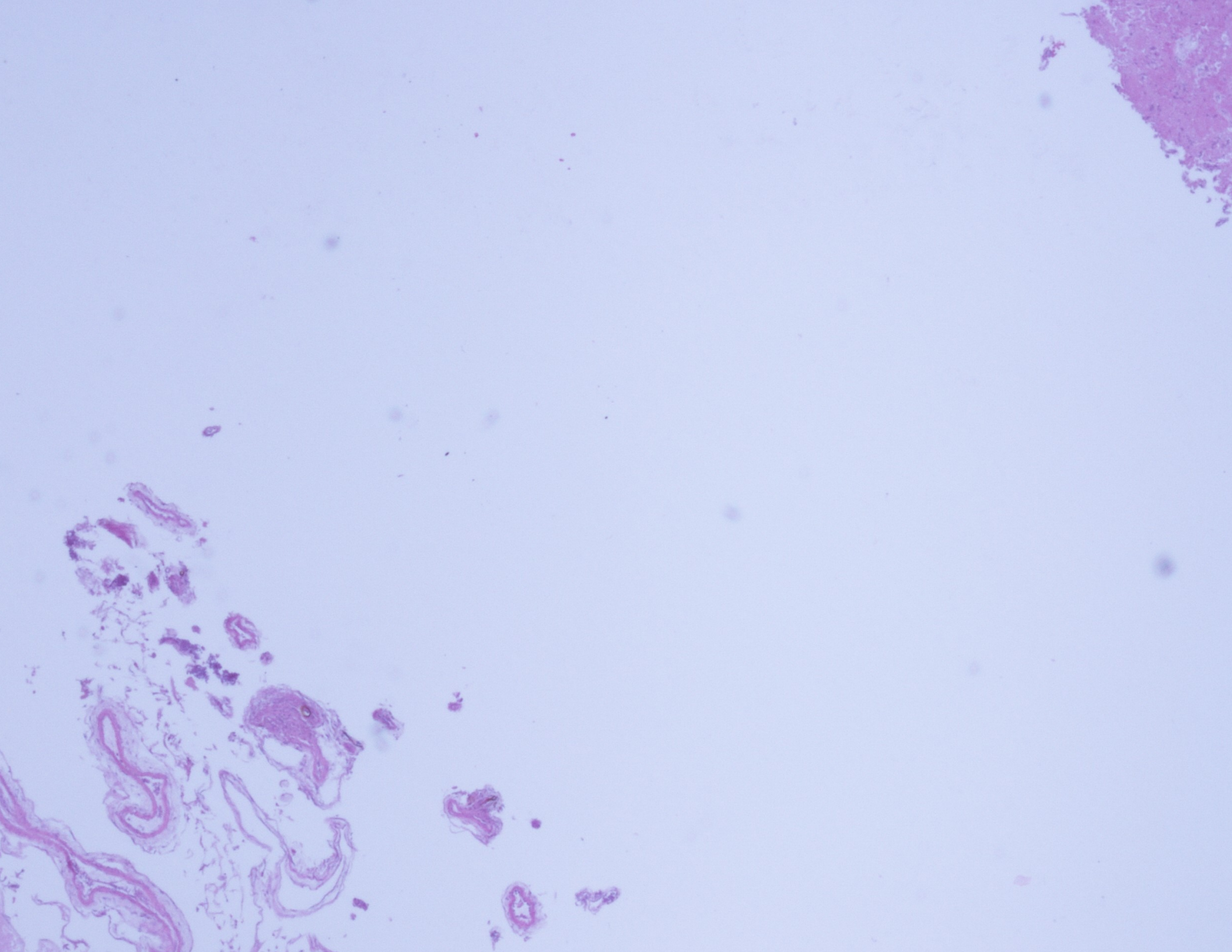

Supplement: S10 Fig — H&E 10x. (PDF) [file pone.0167849.s010.pdf]

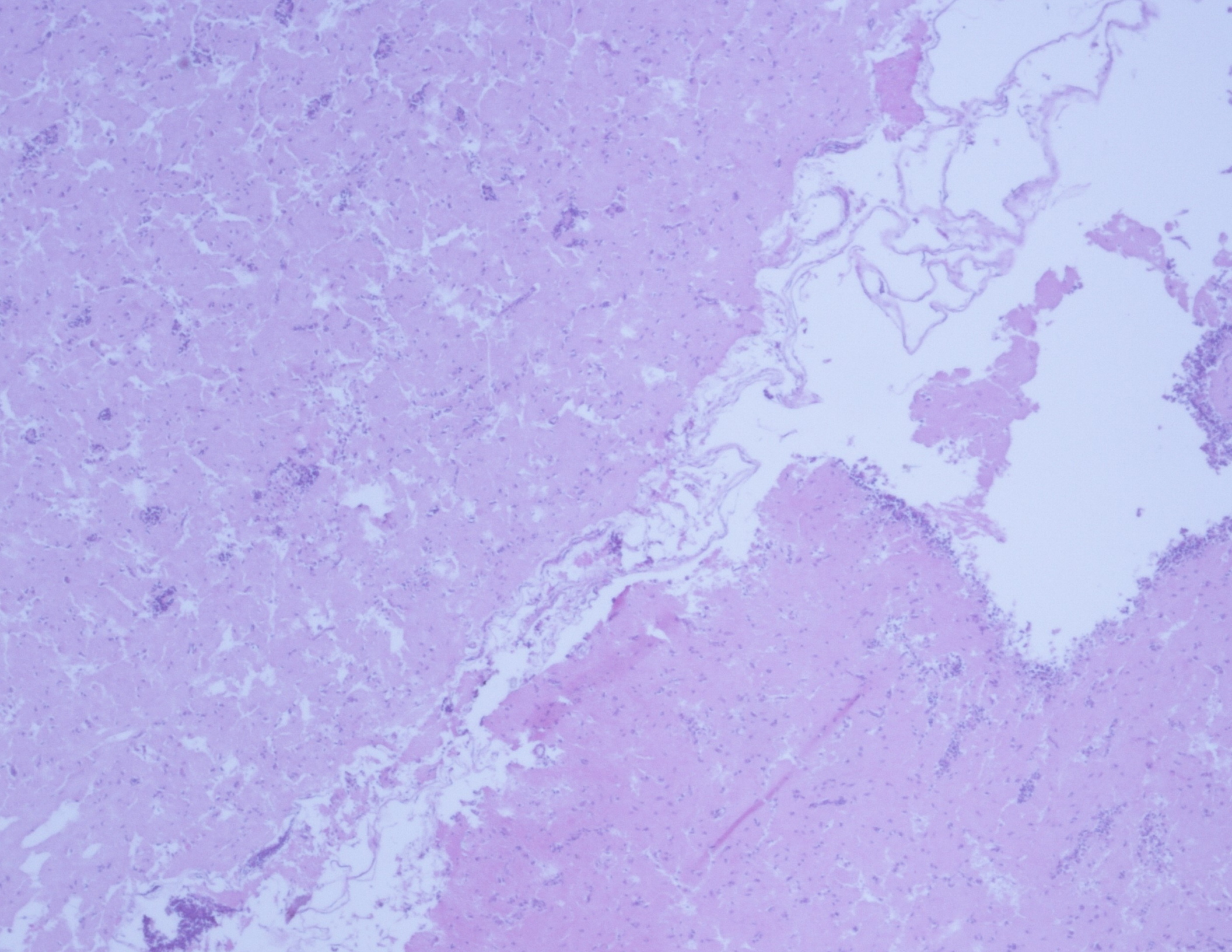

Supplement: S11 Fig — H&E 10x. (PDF) [file pone.0167849.s011.pdf]

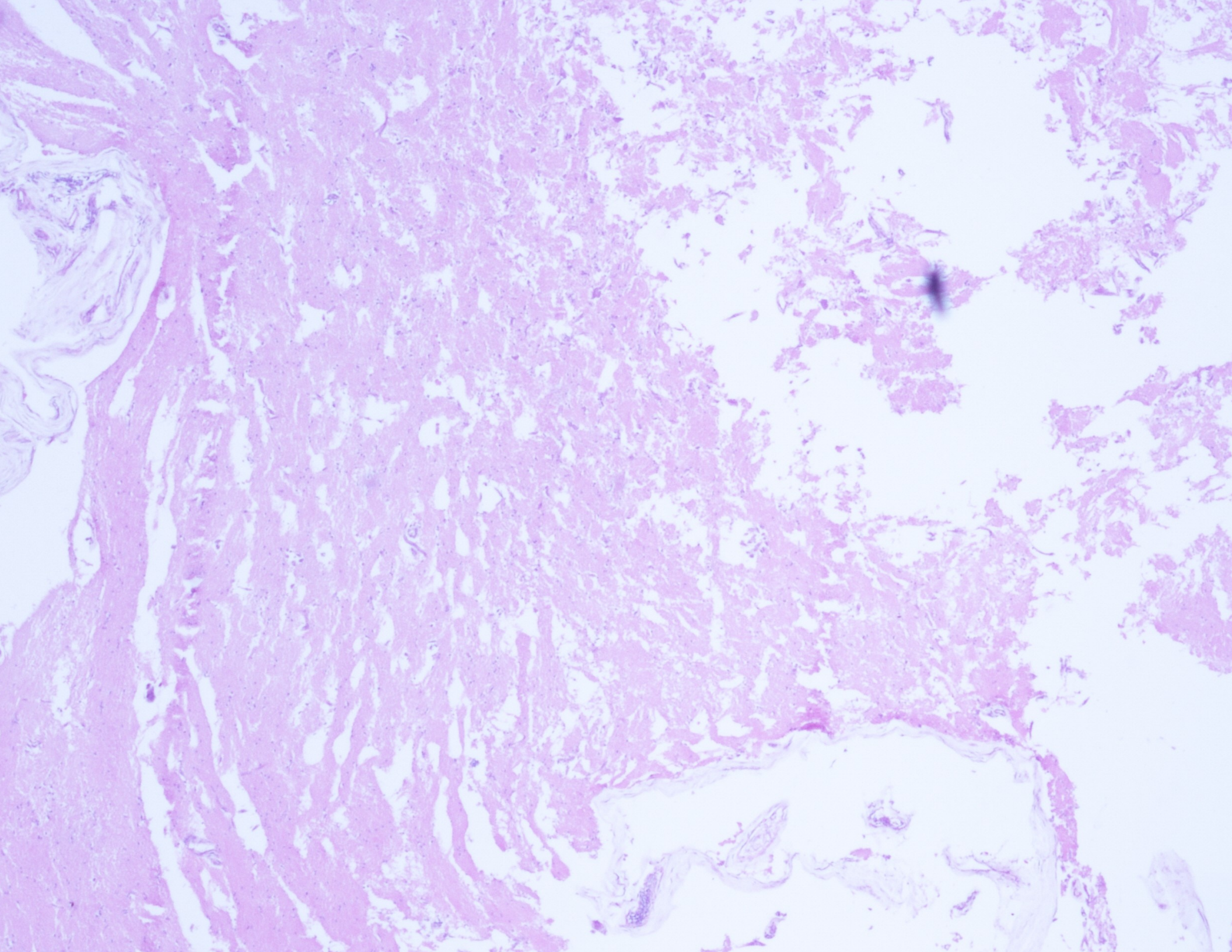

Supplement: S12 Fig — H&E 10x. (PDF) [file pone.0167849.s012.pdf]

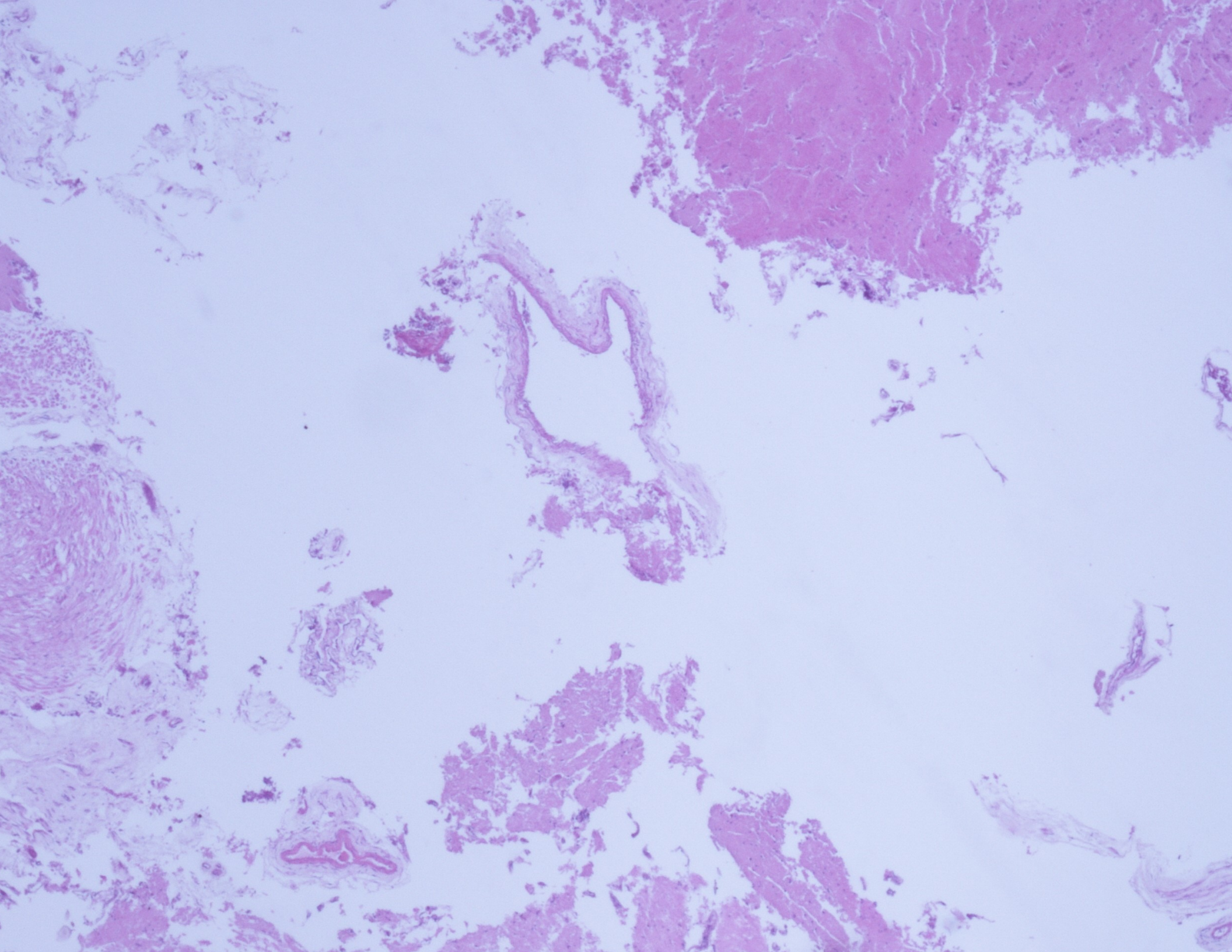

Supplement: S13 Fig — H&E 10x. (PDF) [file pone.0167849.s013.pdf]

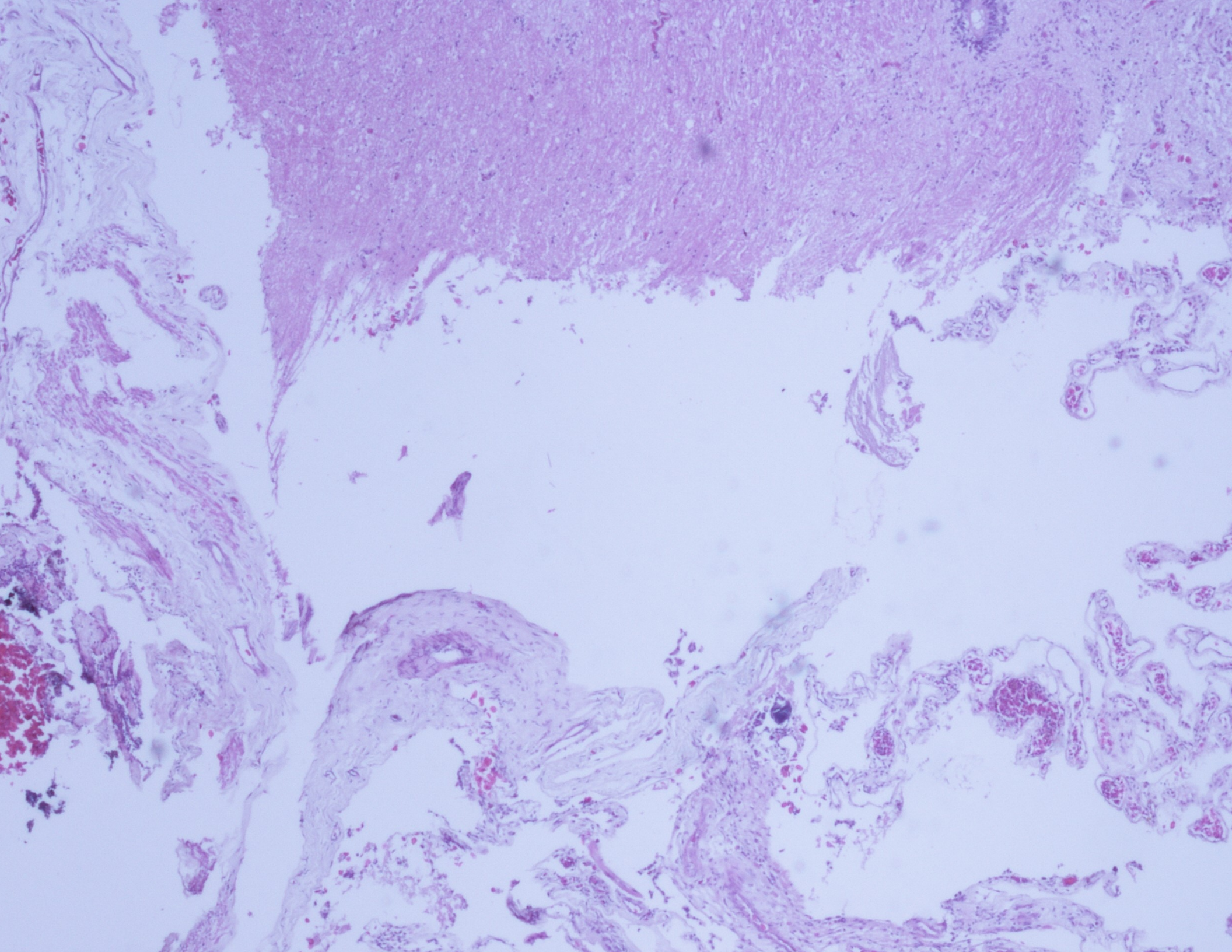

Supplement: S14 Fig — H&E 10x. (PDF) [file pone.0167849.s014.pdf]

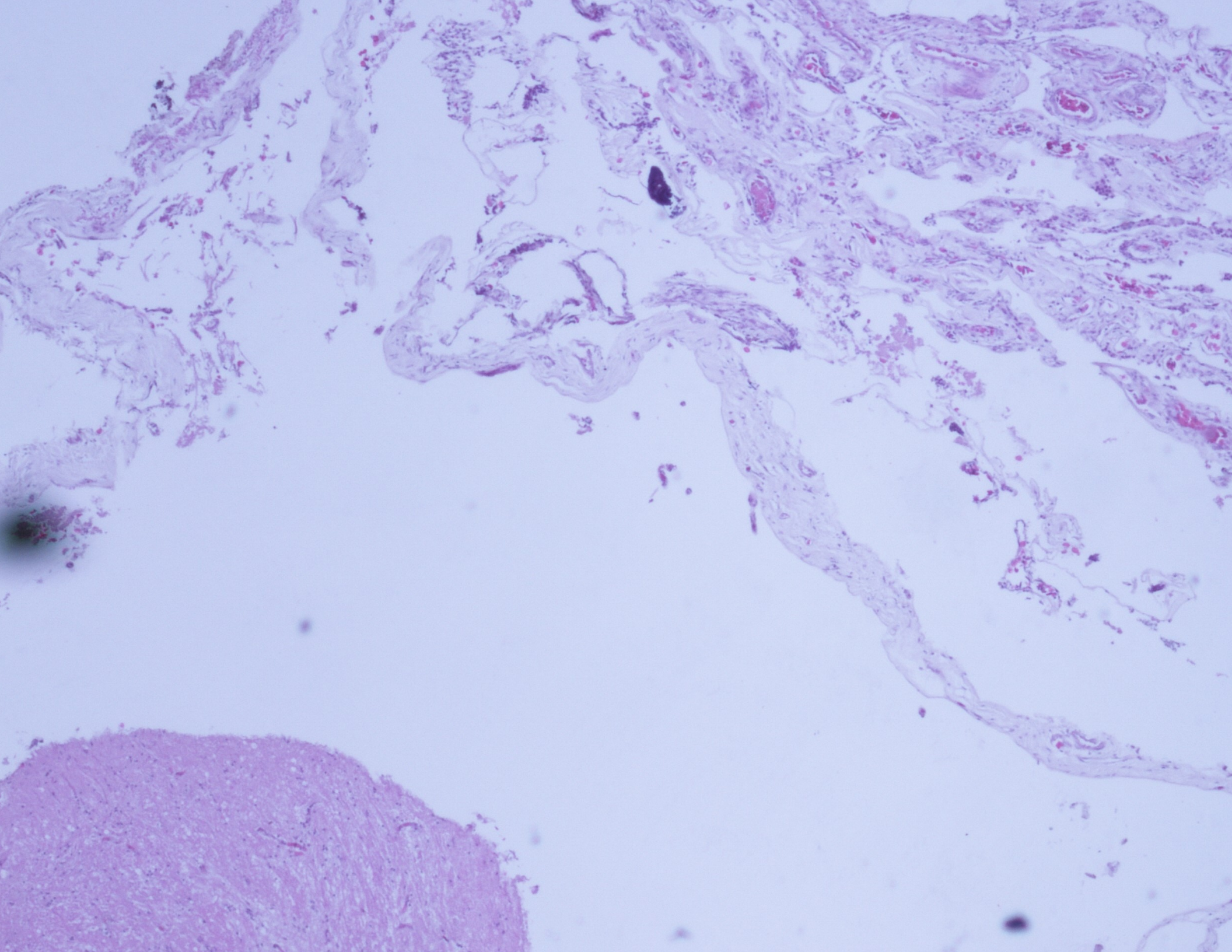

Supplement: S15 Fig — H&E 10x. (PDF) [file pone.0167849.s015.pdf]

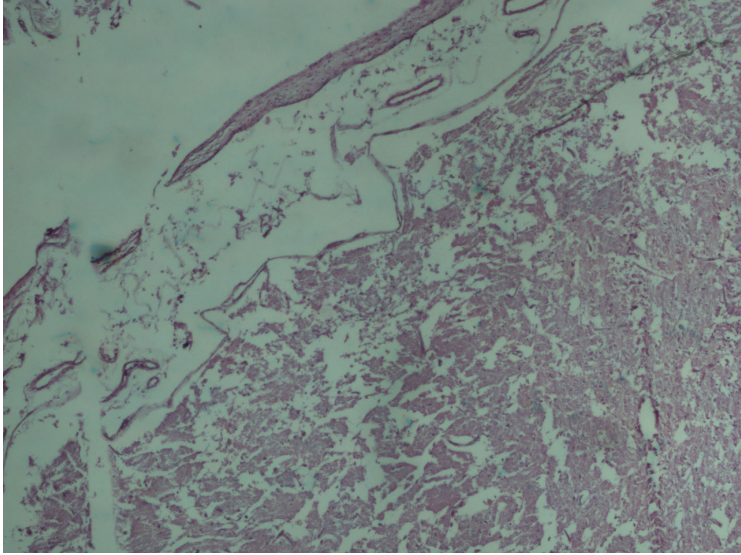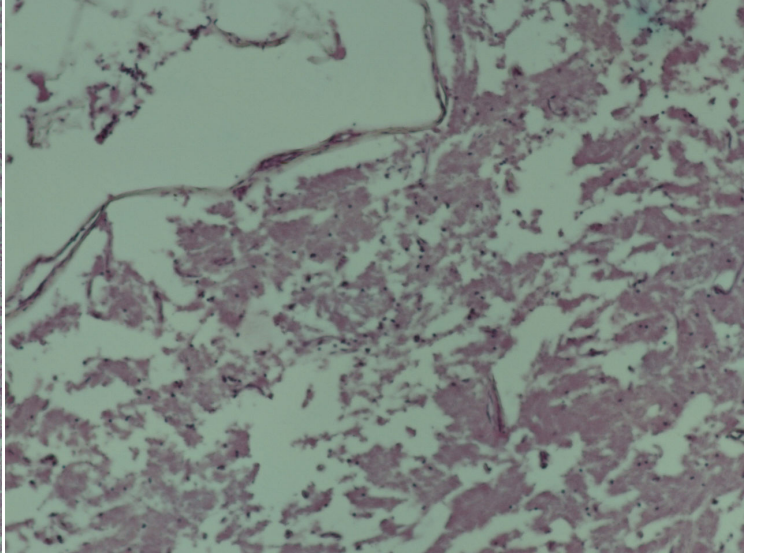

13/0900

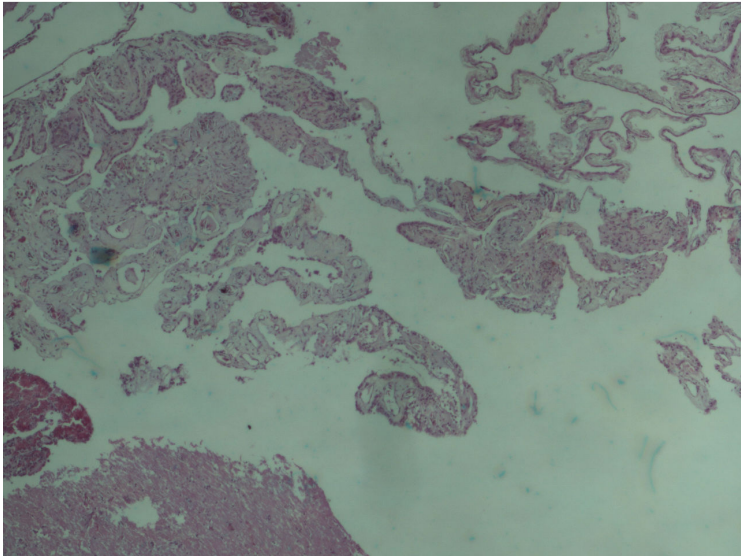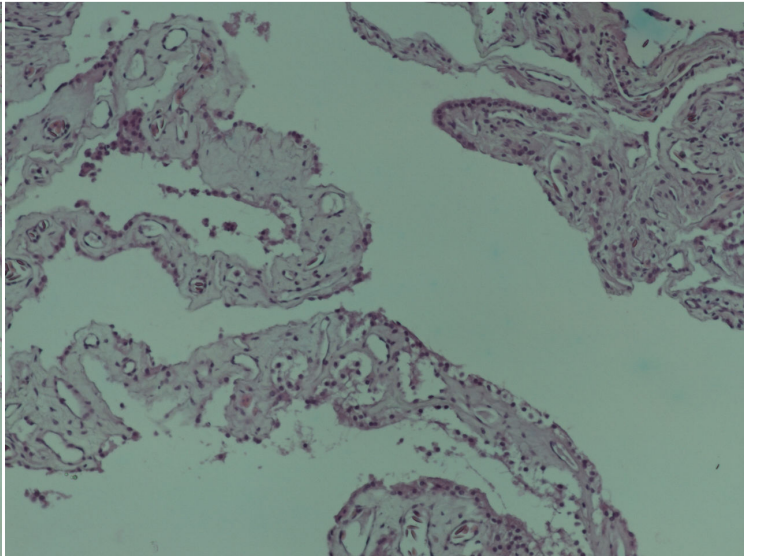

13/0901

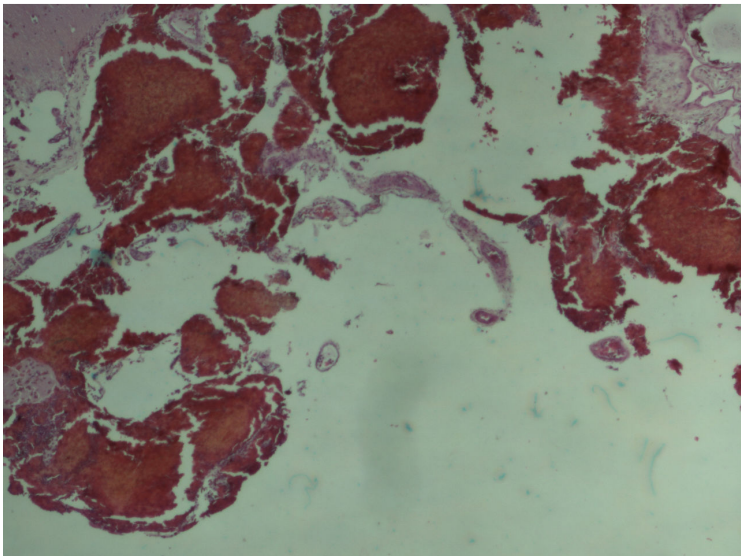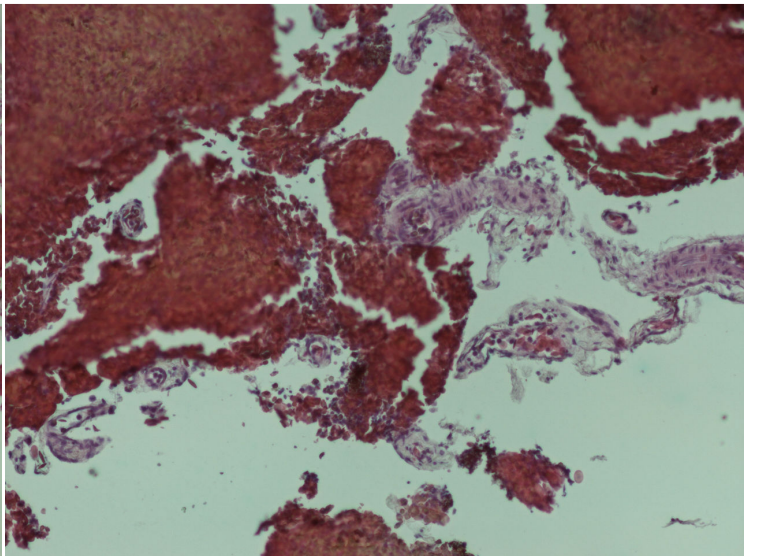

13/0902

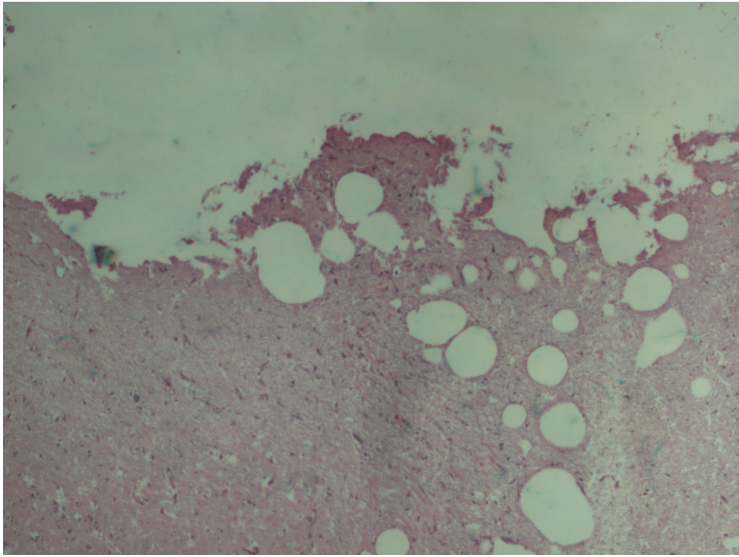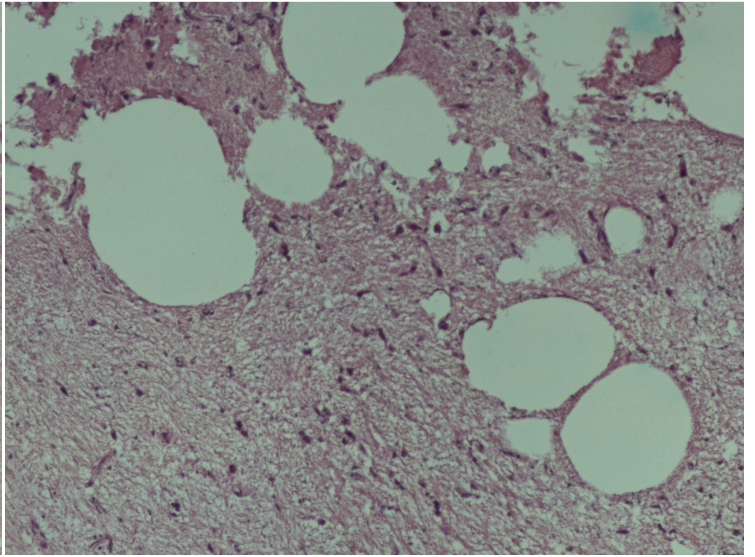

13/0903

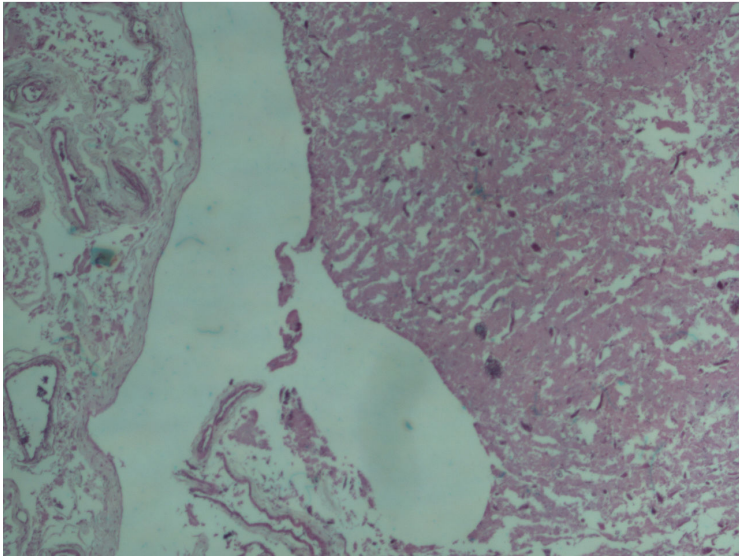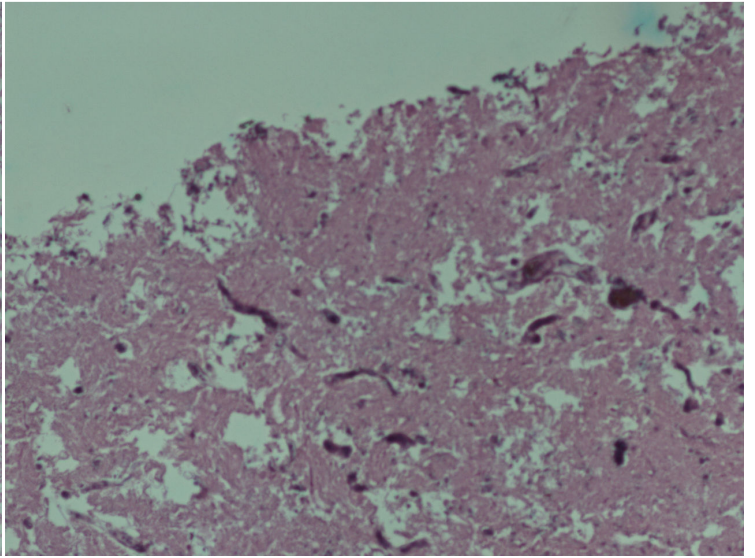

13/0904

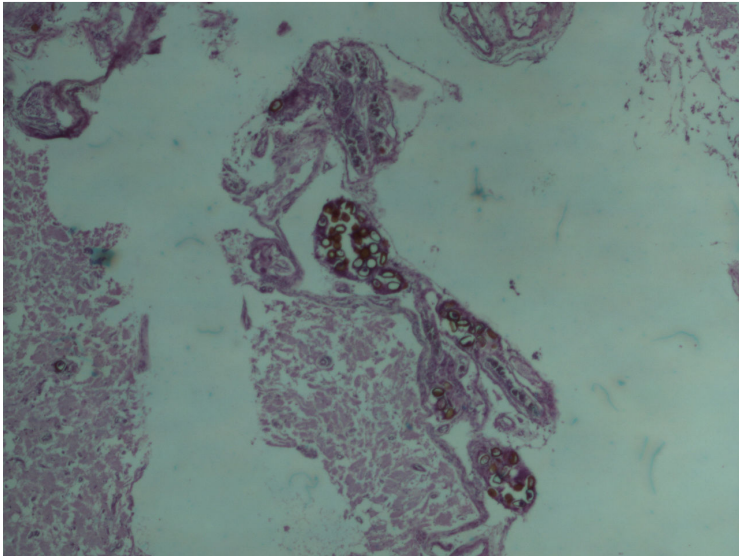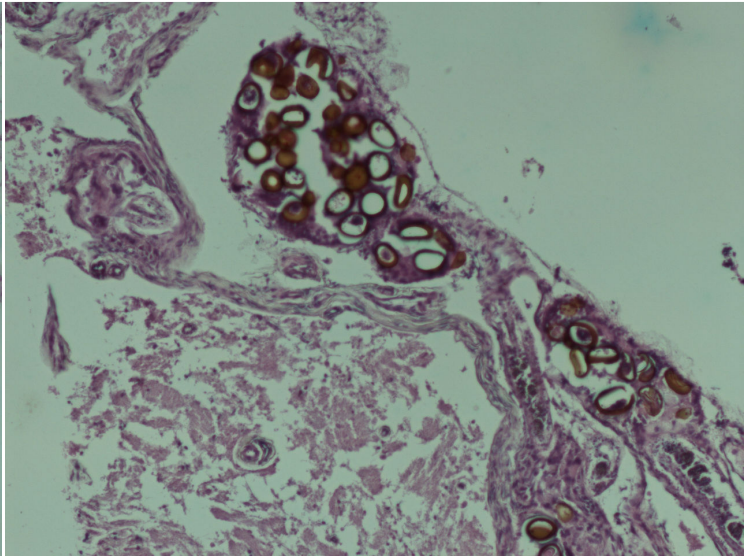

13/0905

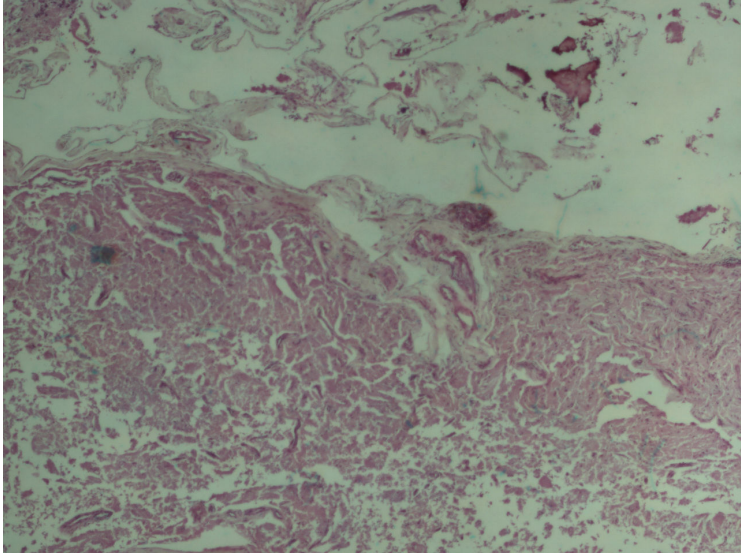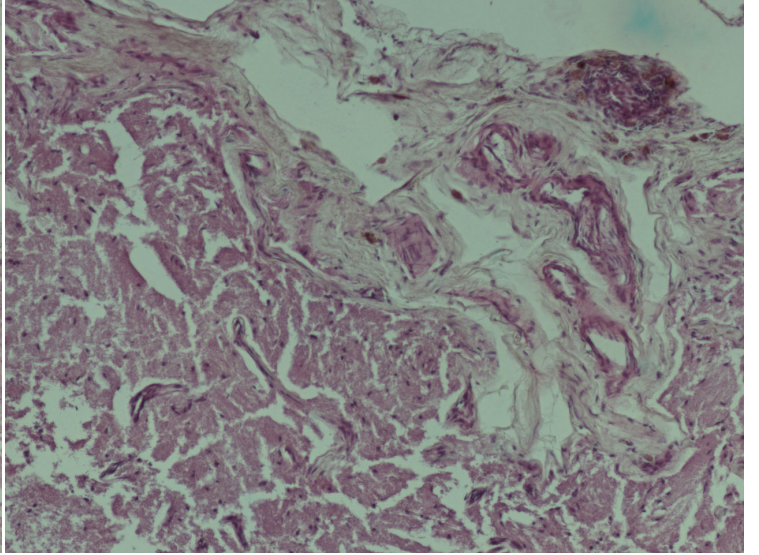

13/0906

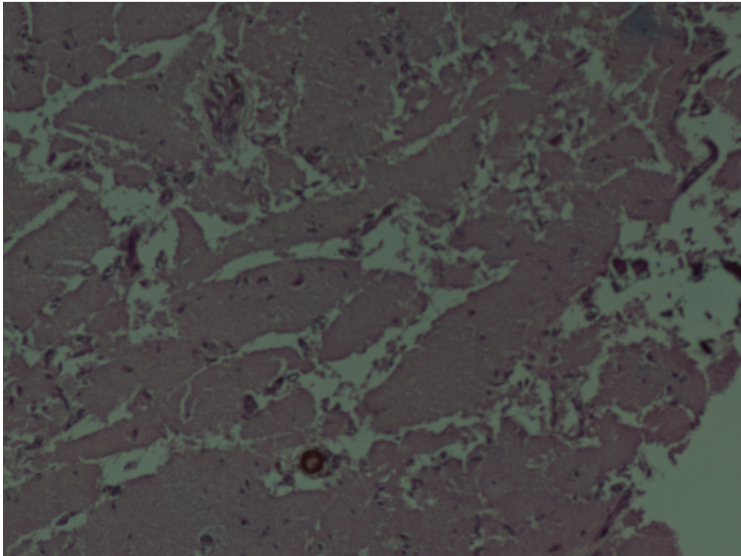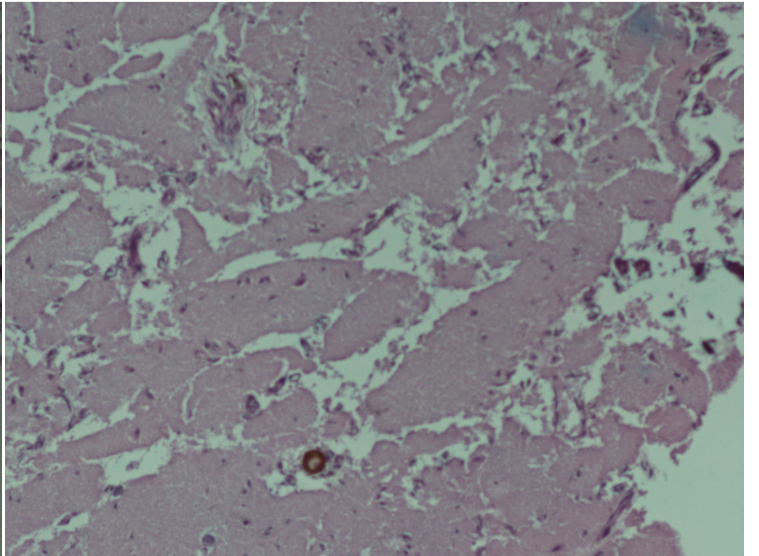

13/0907

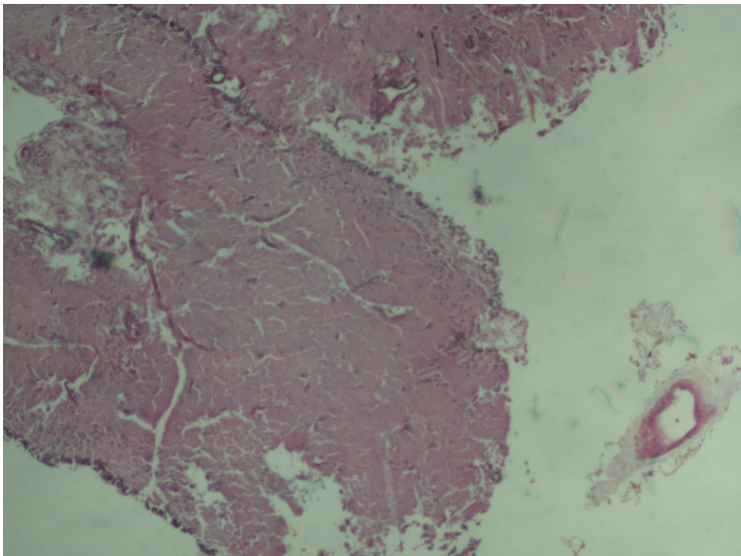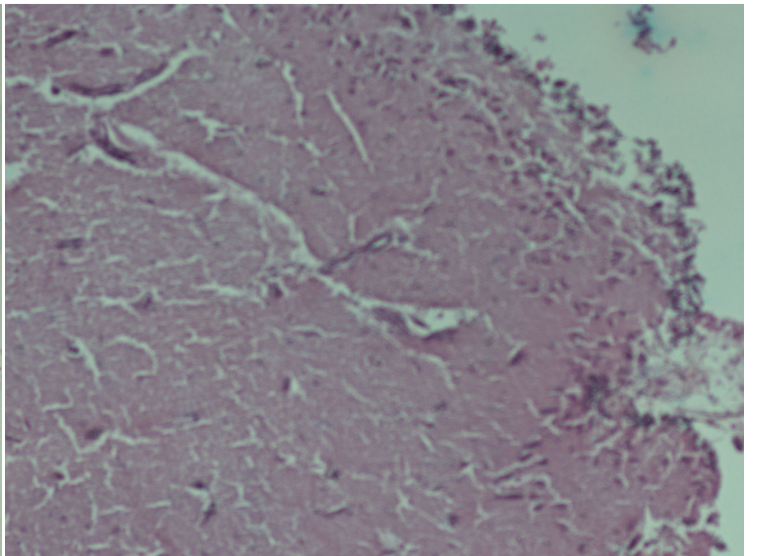

13/0908

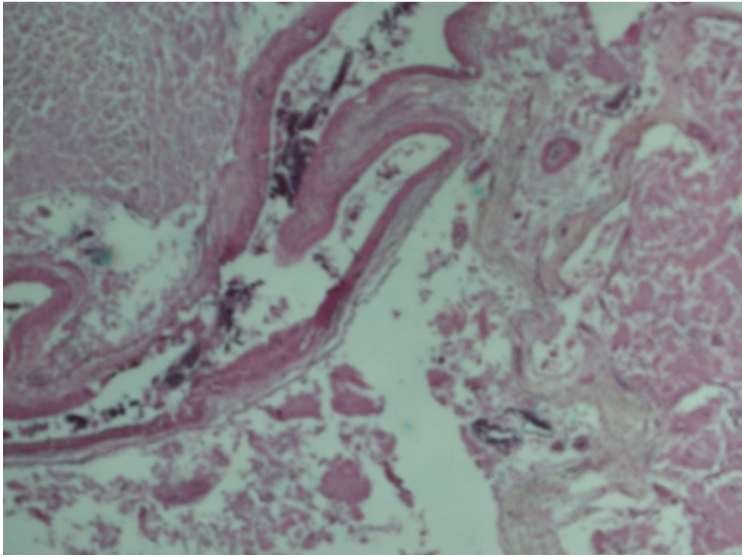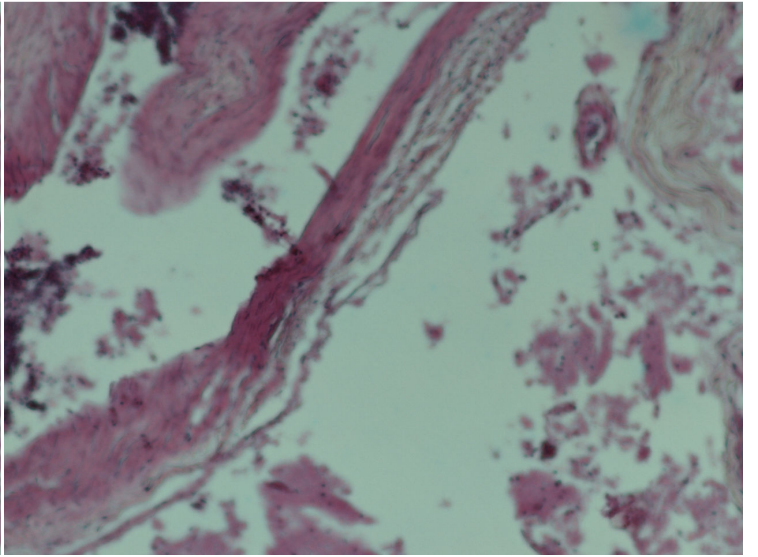

13/0909

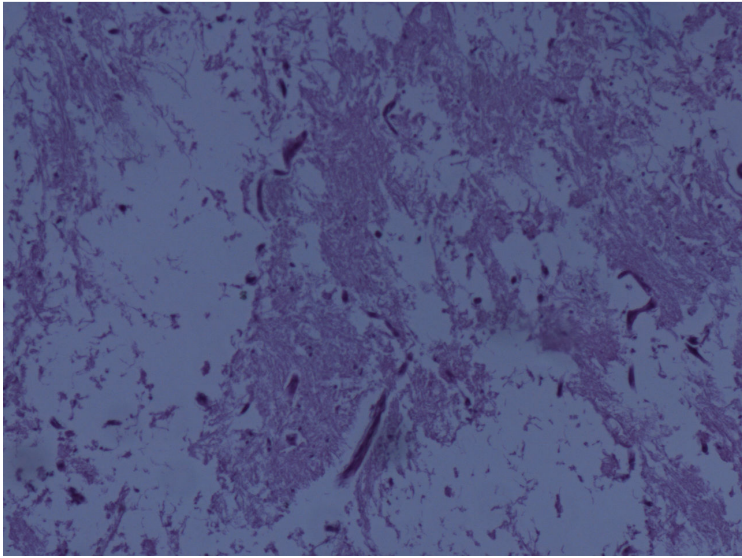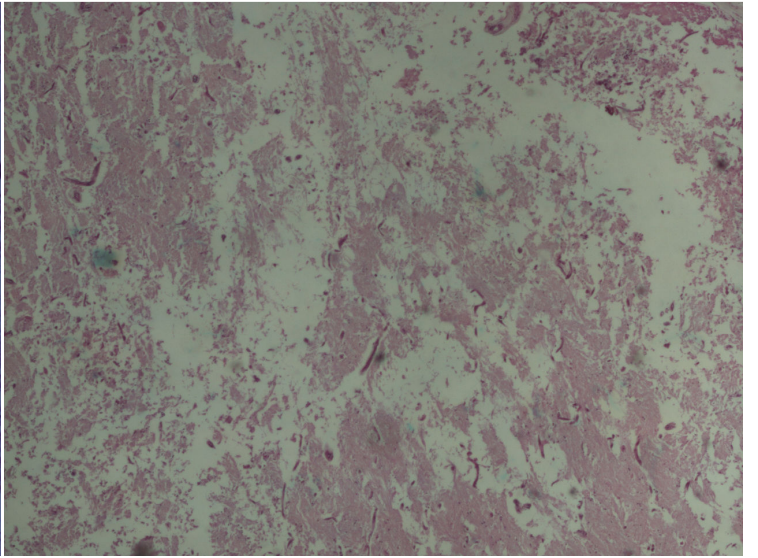

13/0910

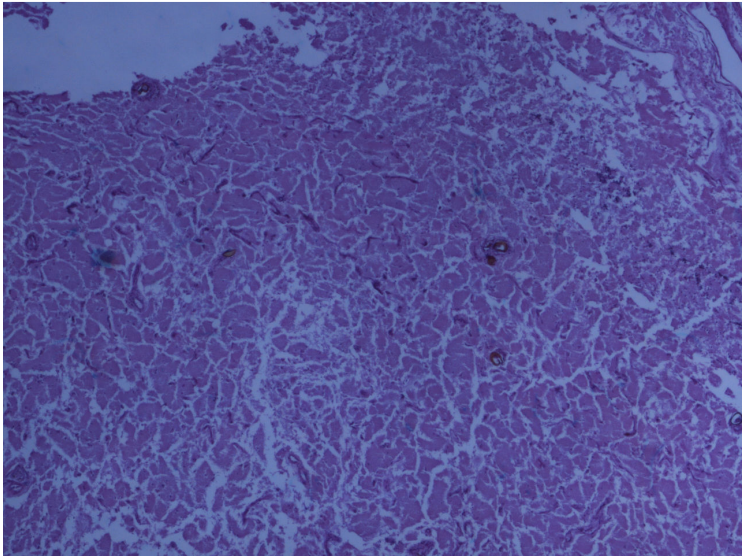

13/0911

Supplement: S16 Fig — H&E 10x. (PDF) [file pone.0167849.s016.pdf]
